# Supplementary material for: Single-atom Pt in intermetallics as an ultrastable and selective catalyst for propane dehydrogenation
Source: Nat Commun. 2020 Jun 5;11:2838. doi: 10.1038/s41467-020-16693-9 (PMC7275083; doi:10.1038/s41467-020-16693-9)
Supplement: Supplementary file 1 — Supplementary Information [file 41467_2020_16693_MOESM1_ESM.pdf]

Supplementary Information

Single-atom Pt in intermetallics as an ultrastable and selective catalyst for propane dehydrogenation

Nakaya *et al*.

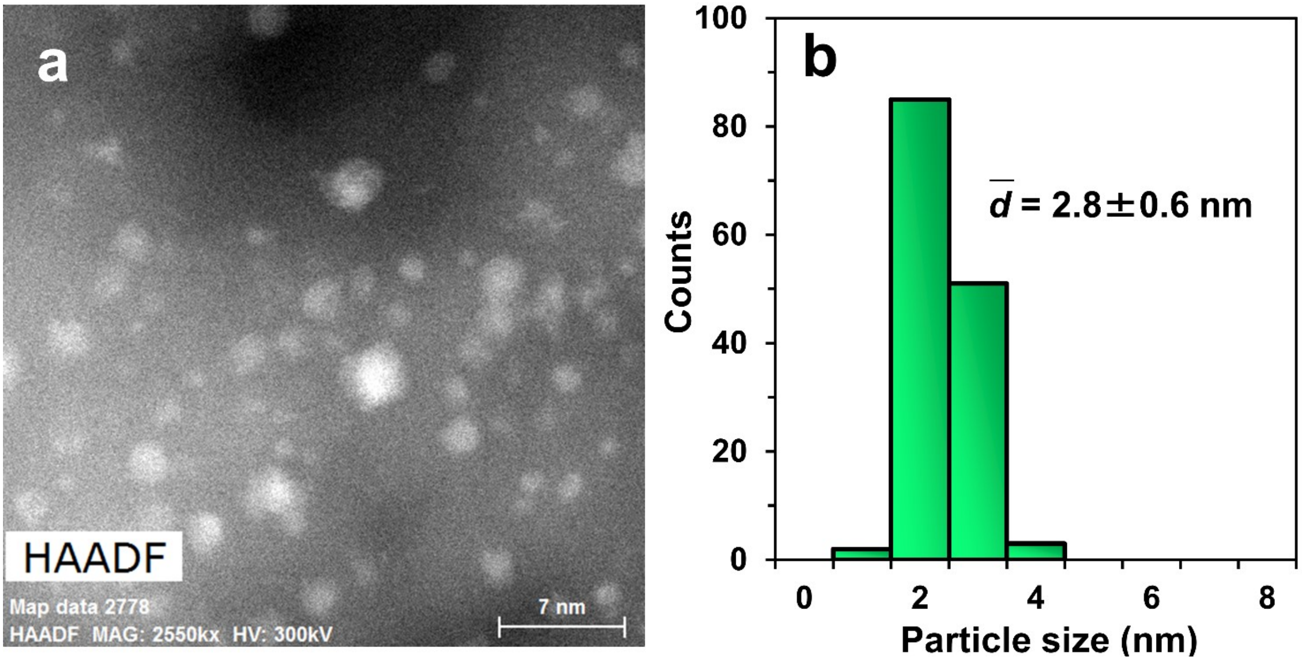

Supplementary Figure 1. a High angle annular dark field scanning transmission electron microscopy (HAADF-STEM) image of PtGa-Pb/SiO_2 (Pt/Pb = 2) and **b** the size distribution of nanoparticles. The particle size distribution was narrow with small and uniform nanoparticles. The particle sizes ranged from 1 nm to 4 nm with a volume weighted average of 2.8 ± 0.6 nm.

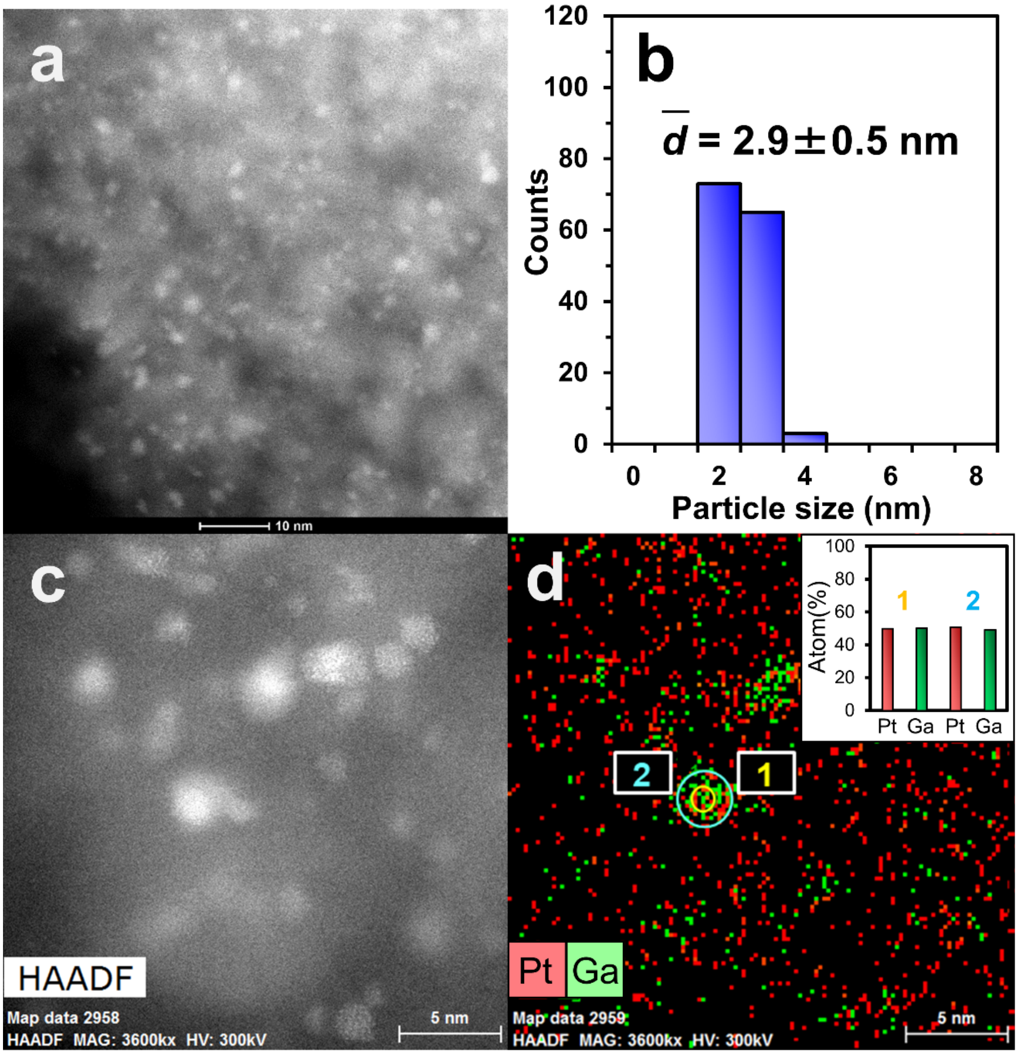

Supplementary Figure 2. a HAADF-STEM image of PtGa/SiO_2 and **b** the size distribution of nanoparticles. **c** HAADF-STEM image of PtGa/SiO_2 and **d** the corresponding elemental map of the Pt + Ga overlayer obtained using energy-dispersive X-ray (EDX) analyzer. Enclosed areas with a yellow and blue circles are correspond to area 1 (core of the nanoparticle) and area 2 (overall of the nanoparticle), respectively.

Supplementary Note 1. Structural analysis of PtGa/SiO_2: Supplementary Figures 2a and b show the HAADF-STEM image of PtGa/SiO_2 and the particle size distribution, respectively. The particle sizes ranged from 1 nm to 4 nm with a volume weighted average of 2.9 ± 0.5 nm. Supplementary Figures 2c and d shows the narrow HAADF-STEM image of PtGa/SiO_2 and the elemental maps of Pt + Ga overlayer acquired using EDX analysis, respectively. The atomic ratios of Pt/Ga in a nanoparticle were 1.0 in both of Area 1 (core) and Area 2 (overall), that consist with the identical ratio of intermetallic PtGa.

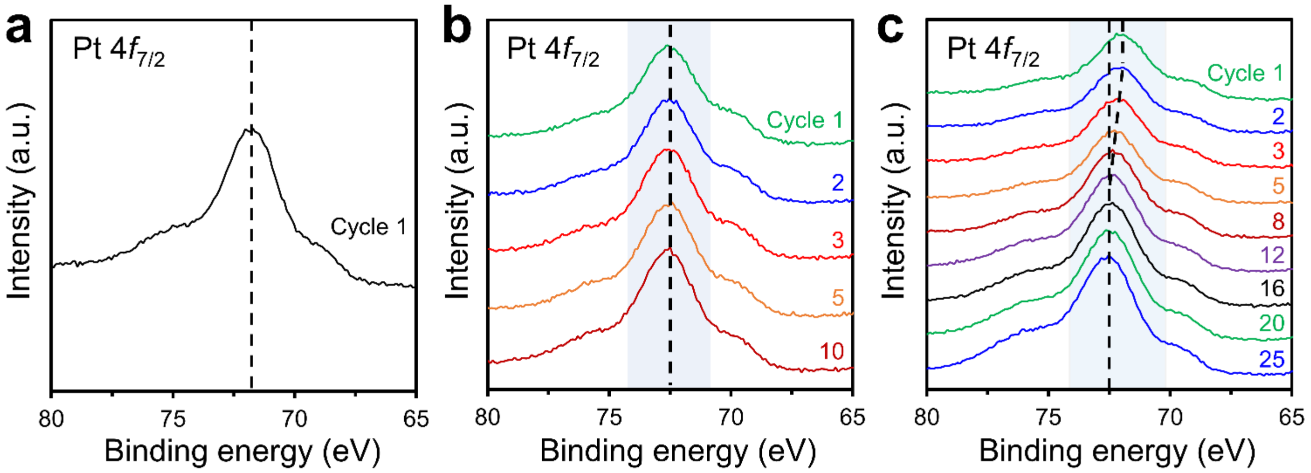

Supplementary Figure 3. Binding energy of Pt 4$f_{7/2}$ electrons obtained by X-ray photoelectron spectroscopy (XPS); **a** Pt/SiO_2, **b** PtGa/SiO_2, and **c** PtGa-Pb/SiO_2 (Pt/Pb = 2). The surface of the sample was sputtered by Ar^+ ion (voltage: 400V, rate: 20%, time: 1 s) at each cycle.

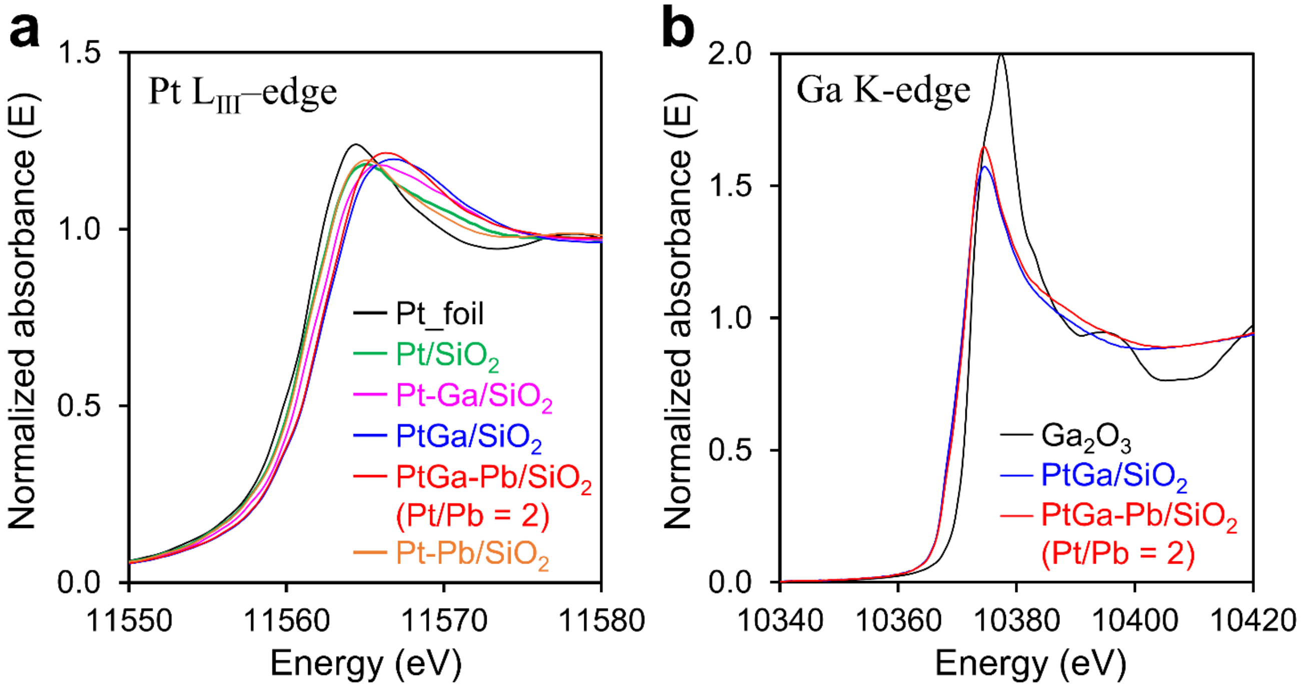

Supplementary Figure 4. **a** Pt L_{III}- and **b** Ga K-edge X-ray absorption near edge structure (XANES) spectra of the reduced catalysts and reference compounds. The atomic ratio of Pt/M in Pt-M/SiO_2 (M = Ga and Pb) was 3.

Supplementary Note 2. XANES spectra of the catalysts: the Pt L_{III} absorption edges and white line heights of Pt-Ga/SiO_2 (Pt/Ga = 3) and PtGa/SiO_2 were, respectively, shifted to higher energy and slightly increased compared with those of Pt/SiO_2, indicating that the electron density of Pt decreased upon alloying with Ga. This is consistent with the result of the comprehensive XANES and DFT studies for intermetallic $PtGa_2$.[1] On the contrary, for PtGa-Pb/SiO_2 (Pt/Pb = 2), the absorption edge was slightly shifted to lower energy compared with PtGa/SiO_2, indicating that the electron density of Pt metal increased by Pb modification (Fig. 2). Similar electron donation by Pb was also reported for other Pt-Pb bimetallic systems.[2] For the Ga K-edge XANES spectra, the adsorption edges of PtGa and PtGa-Pb were much lower in energy than that of Ga_2O_3, indicating the presence of metallic Ga. However, the white line intensities of them were somewhat high, which suggests that a part of Ga is oxidized. According to the literature by Copéret *et al*. showing that Ga in Pt-Ga was completely reduced under flowing H_2 at 550°C,[3] the presence of oxidized Ga might be attributed to aerobic oxidation of a part of surface Ga by sub-ppm-level contaminated oxygen in the grove box during sample preparation. This is consistent with the high oxophilic character of Ga.[4,5]

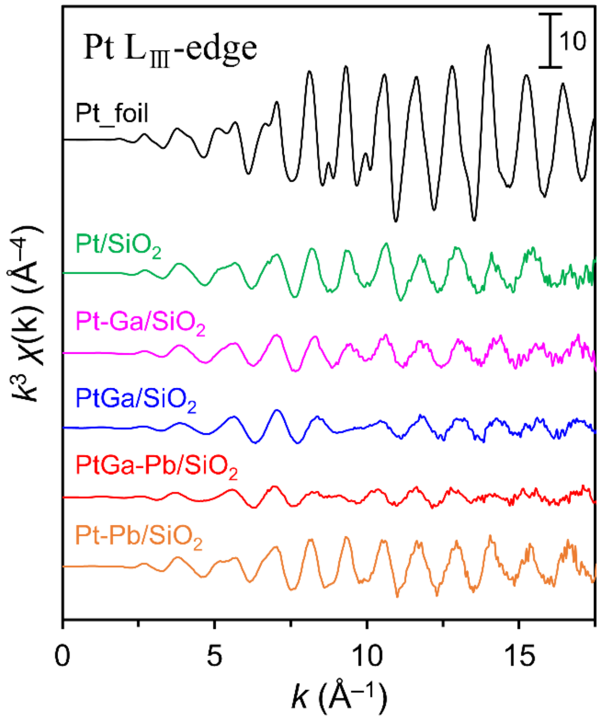

Supplementary Figure 5. Pt L$_{III}$-edge k^3-weighted raw extended X-ray absorption fine structure (EXAFS) oscillations for silica-supported Pt-based catalysts and Pt foil: Pt foil (Black), Pt/SiO_2 (Green), Pt-Ga/SiO_2 (Pt/Ga = 3) (Pink), PtGa/SiO_2 (Blue), PtGa-Pb/SiO_2 (Pt/Pb = 2) (Red), Pt-Pb/SiO_2 (Pt/Pb = 3) (Orange).

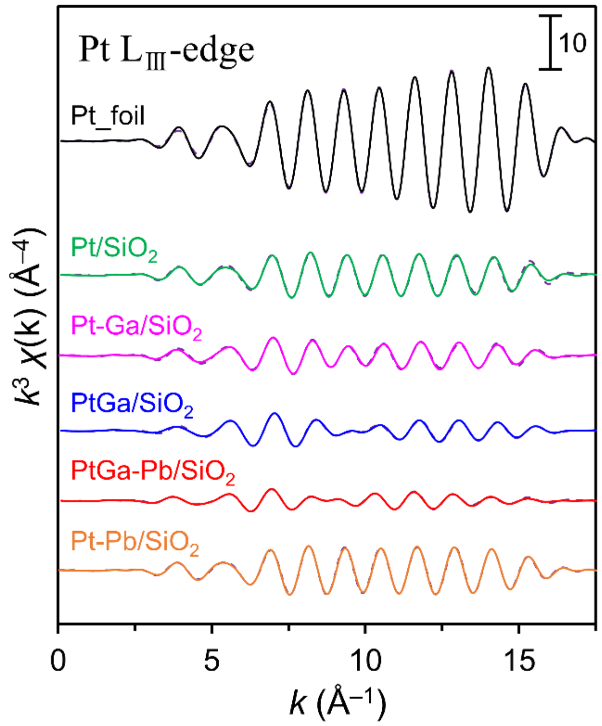

Supplementary Figure 6. Curve-fitting results of Pt L$_{III}$-edge k^3-weighted EXAFS for silica-supported Pt-based catalysts and Pt foil: Pt foil (Black), Pt/SiO_2 (Green), Pt-Ga/SiO_2 (Pt/Ga = 3) (Pink), PtGa/SiO_2 (Blue), PtGa-Pb/SiO_2 (Pt/Pb = 2) (Red), Pt-Pb/SiO_2 (Pt/Pb = 3) (Orange). Solid and dashed lines indicate the results of simulation and experiment, respectively.

Supplementary Table 1. Summary of the Pt L_{III}-edge k^3-weighted EXAFS curve fitting for Pt-based catalysts and reference Pt foil.

Sample	Shell	S_o^{2}[a]	*CN*[b]	*R* (Å)[c]	ΔE_o (eV)[d]	σ^2 ($Å^2$)[e]	R-factor (R^2)
Pt_foil	Pt–Pt	0.91	12.0 (fix)	2.77 ± 0.00	4.8 ± 0.4	0.005 ± 0.000	0.002
Pt/SiO_2	Pt–Pt	0.91	7.0 ± 0.4	2.73 ± 0.00	3.9 ± 0.7	0.007 ± 0.000	0.009
Pt-Ga/SiO_2	Pt–Ga	0.91	1.6 ± 1.1	2.50 ± 0.01	−1.9 ± 1.3	0.013 ± 0.005	0.015
(Pt/Ga = 3)	Pt–Pt	0.91	5.2 ± 0.8	2.71 ± 0.01		0.008 ± 0.001	
PtGa/SiO_2	Pt–Ga	0.91	2.6 ± 0.5	2.49 ± 0.01	−4.4 ± 1.1	0.011 ± 0.001	0.010
	Pt–Pt	0.91	2.7 ± 0.4	2.70 ± 0.01		0.007 ± 0.001	
PtGa-Pb/SiO_2	Pt–Ga	0.91	1.8 ± 0.3	2.50 ± 0.01		0.010 ± 0.001	
	Pt–Pt	0.91	4.5 ± 1.3	2.75 ± 0.01	−1.7 ± 2.0	0.010 ± 0.001	0.007
	Pt–Pb	0.91	1.0 ± 1.0	2.88 ± 0.03		0.012 ± 0.006	
Pt-Pb/SiO_2 (Pt/Pb = 3)	Pt–Pt	0.91	7.5 ± 0.3	2.74 ± 0.01	2.3 ± 0.5	0.007 ± 0.000	0.004

[a] Amplitude factor. [b] Coordination number. [c] Distance between absorber and backscatterer atoms. [d] Correction term in the absorption edge. [e] Disorder term (EXAFS Debye–Waller factor).

Supplementary Note 3. EXAFS curve fitting for Pt-based catalysts: PtGa/SiO_2 showed Pt–Ga and Pt–Pt scatterings at 2.49 Å (CN = 2.6) and 2.70 Å (CN = 2.7), respectively. The Pt–Ga scattering at 2.49 Å, which originated from intermetallic PtGa, indicated the formation of intermetallic PtGa compounds. PtGa-Pb/SiO_2 showed Pt–Ga, Pt–Pt, and Pt–Pb scatterings at 2.50 Å (CN = 1.8), 2.75 Å (CN = 4.5), and 2.88 Å (CN = 1.0), respectively (Supplementary Table 1). The Pt–Ga scattering at 2.50 Å, which originated from intermetallic PtGa, indicated the formation of intermetallic PtGa compounds. The small contribution of Pt–Pb scattering (CN = 1.0) was observed, which indicates the presence of Pb atoms with close contact to Pt. Pt-Ga/SiO_2 (Pt/Ga =3) showed Pt–Ga and Pt–Pt scatterings at 2.50 Å (CN = 1.6) and 2.71 Å (CN = 5.2), respectively. The Pt–Ga scattering at 2.49 Å, which originated from intermetallic PtGa, indicated the formation of intermetallic PtGa compounds. Considering that the higher Pt–Pt scattering and diffraction angles (Supplementary Figure 16), which consistent with those of monometallic Pt, Pt-Ga/SiO_2 (Pt/Ga =3) likely has Pt-PtGa core-shell structure.

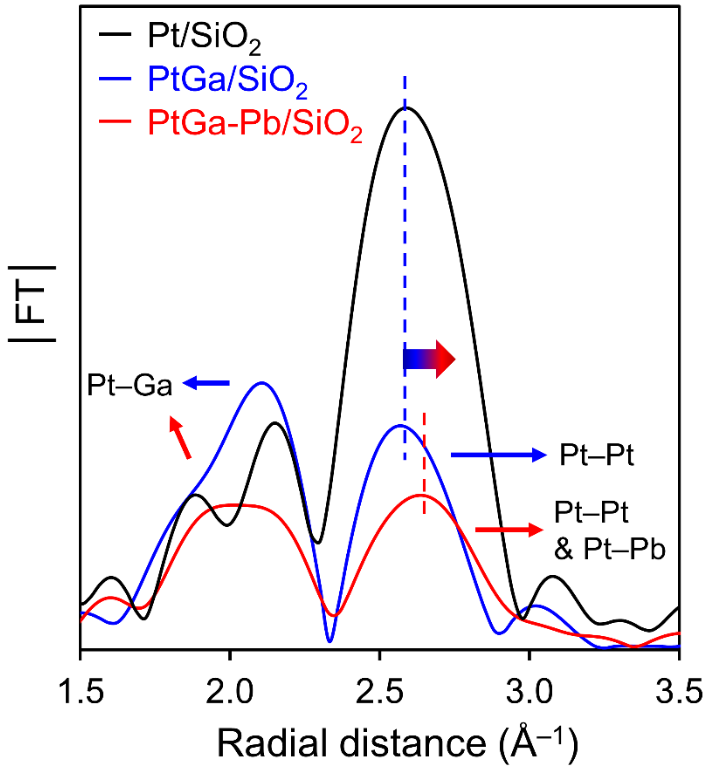

Supplementary Figure 7. Magnitude of Fourier transform of the k^3-weighted EXAFS spectra of reduced catalysts. Δk = 3–16 Å.

Supplementary Table 2. Pt dispersions of silica-supported Pt-based catalysts.[a]

Catalyst	Dispersion of Pt (%)
Pt	37.1
Pt-Ga (Pt/Ga = 3)	24.9
PtGa	9.9
Pt_3Sn	24.2
PtSn	8.0
Pt_3In	20.2
Pt-Pb (Pt/Pb = 3)	17.5
PtGa-Pb (Pt/Pb = 5)	8.5
PtGa-Pb (Pt/Pb = 2.5)	4.6
PtGa-Pb (Pt/Pb = 2)	5.9
PtGa-Pb (Pt/Pb = 1.5)	2.5
PtGa-Sn (Pt/Sn = 2)	9.8
PtGa-In (Pt/In = 2)	6.4

[a] The loading amount of Pt is fixed at 3 wt% for all the catalysts.

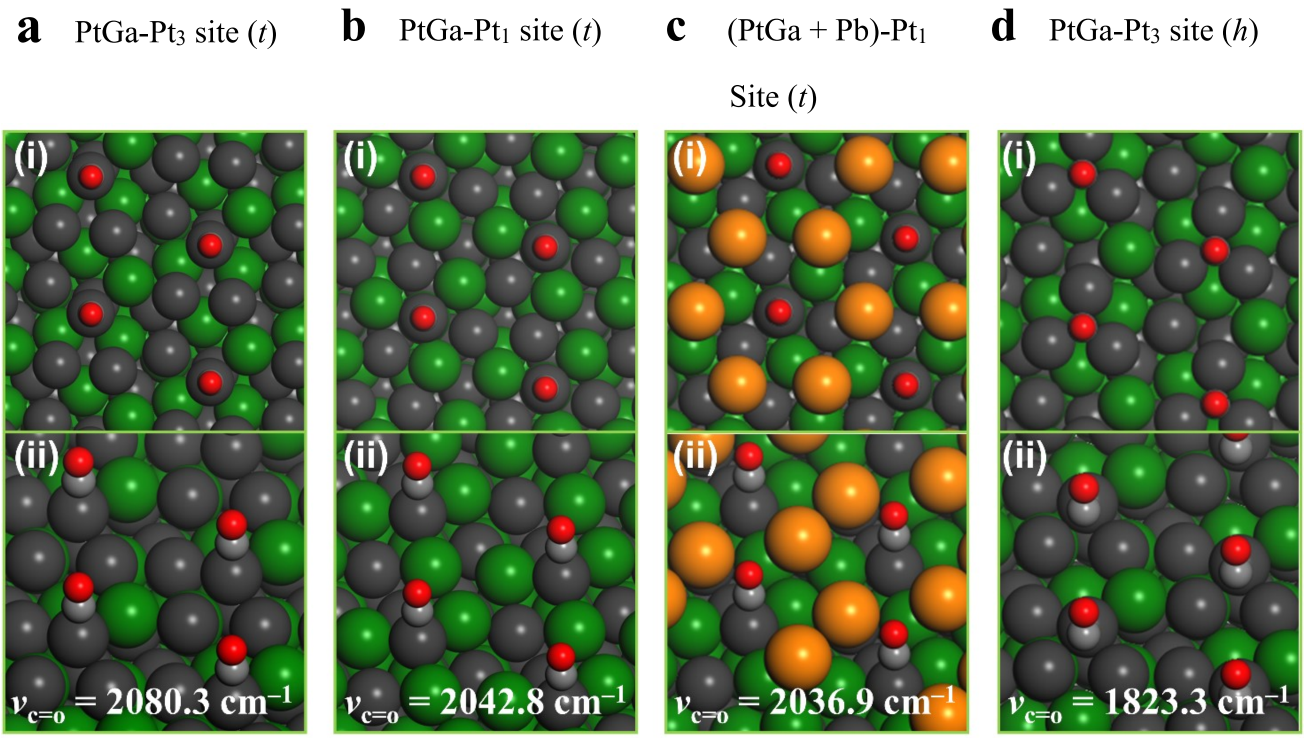

Supplementary Figure 8. Optimized structures of CO adsorbed on **a** PtGa-Pt_3 (*t*), **b** PtGa-Pt_1 (*t*), **c** (PtGa + Pb)-Pt_1 (*t*), and (d) PtGa-Pt_3 (*h*). Adsorption geometries of on-top and three-fold hollow CO were abbreviated as (*t*) and (*h*), respectively. (i) Upper and (ii) lower figures indicate top and diagonal views, respectively. Black: Pt, green: Ga, orange: Pb, gray: C, red: O.

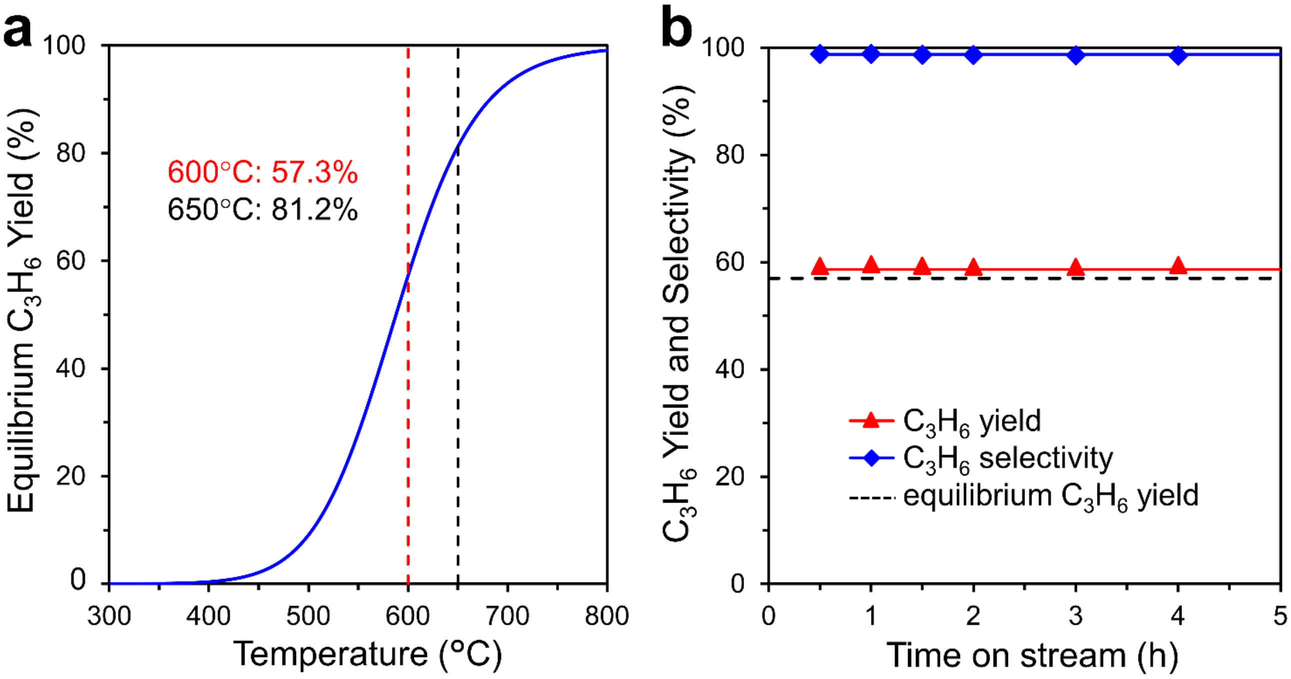

Supplementary Figure 9. **a** Simulated (600 and 650°C) and **b** experimental (600°C) equilibrium C_3H_6 yield in the reaction condition used in this study. Good agreement was obtained between the simulation and experiment. Excess amount of PtGa-Pb/SiO_2 (150 mg) was used so that C_3H_6 yield reaches equilibrium.

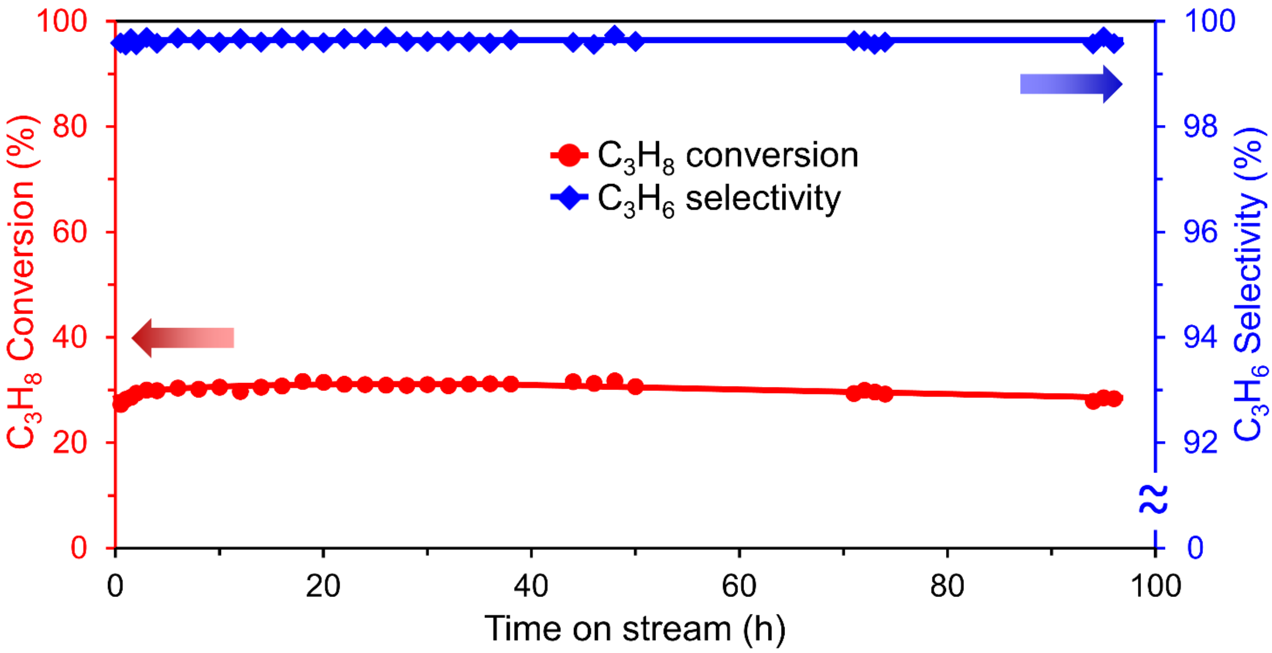

Supplementary Figure 10. Long-term stability test in PDH on PtGa-Pb/SiO_2 (Pt/Pb = 2) at 600°C.

Supplementary Note 4. First-order deactivation model:

The first-order deactivation model was used to estimate the catalytic stability.[6] k_d (h^{-1}) and τ (h) were defined by the following equation. Here, lower k_d and higher τ values are indicative of higher stability.

$$k_\mathrm{d} = \frac{\ln\left(\frac{1 - conv._{end}}{conv._{end}}\right) - \ln\left(\frac{1 - conv._{start}}{conv._{start}}\right)}{t} \tag{1}$$

$$\tau = \frac{1}{k_d} \tag{2}$$

where, $conv._{start}$ and $conv._{end}$ indicates initial and final propane conversion, respectively. t represents the reaction time. k_d (h^{-1})[6] and τ (h) represent the deactivation rate constant and expected catalyst life, respectively.

Supplementary Table 3. Summary of the catalytic data of PtGa-Pb and other reported Pt-based catalysts for PDH.

entry	catalyst	Pt (wt%)	temp. (°C)	conversion (%)[a]	C_3H_6 Selectivity (%)[b]	specific activity (s^{-1})[c]	k_d (h^{-1})[d]	ref
1	PtIn/Mg(Al)O-600	0.6	620	69–50	98	0.45	0.027	7
2	PtIn/Mg(Al)O-x	0.6	620	66.4–43.5	95	0.43	0.118	8
3	0.3PtSn/1.5In-Al	0.3	620	58.4–48.6	93.5	0.75	0.144	9
4	PtGa/SiO_2	3	600	44.7–24.5	98.8	0.56	0.018	This
5	PtGa-Pb/SiO_2 (Pt/Pb = 2)	3	600	30.0 (4 h)–28.4	99.6	0.38	0.001	Study
6	Pt/Mg(Sn)(Al)O	0.5	600	48.3–43.0	86.4	1.05	0.002	10
7	Pt_3In/SiO_2	0.3	600	17.5–17	92	0.08	0.018	11
8	Pt3Ga/CeAl	1	600	41.1–32.2	99.6	0.52	0.026	12
9	Pt3Ga/Al_2O_3	1	600	39.4–28.1	99.5	0.50	0.034	12
10	Pt/CeAl	1	600	47.1–22.2	62	0.59	0.076	12
11	PtSn/TS-1	0.5	600	53.5–47.7	92.5	0.41	0.033	13
12	15%Zn-0.1%/Al_2O_3	–	600	35–31	94	–	0.045	14
13	Pt/TA0	1	600	50.5–21.0	60	0.64	0.134	15
14	Pt/TA10	1	600	47.3–25.9	77	0.60	0.094	15
15	Pt/TA20	1	600	45.5–17.8	89	0.57	0.135	15
16	PtSnIn/08Zr-Al	0.3	600	57.7–51.7	98	0.74	0.097	16
17	0.1Pt10Cu/Al_2O_3	0.1	600	40–22	90	1.97	0.215	17
18	Pt-Sn/SAPO-34-500	0.5	595	34.6–43.9	66	0.48	0.149	18
19	Pt/Al_2O_3 sheet	0.36	590	42.7–15.9	93	1.38	0.058	19
20	PtSn/Al_2O_3 sheet	0.35	590	48.7–44.6	98	1.62	0.007	19
21	Pt-Sn-Na/Al-SBA-15	0.5	590	27.5–12.6	94	0.20	0.024	20
22	Pt-Na/Sn-ZSM-5	0.5	590	41.7–39.1	95.3	0.30	0.011	21
23	PtNa/Zn(1.0%)-ZSM-5	0.5	590	40.6–37.8	93	0.29	0.012	22
24	Pt-Sn/mesoporous Al_2O_3	0.5	590	29.8–24.6	92	0.22	0.044	23
25	Pt-Sn/ZSM-5	0.5	590	33.1–26.3	47.7	0.24	0.054	23
26	Pt-Sn/γ–Al_2O_3	0.5	590	29.4–22.7	76	0.21	0.058	23
27	Pt-Sn/SBA-15	0.5	590	11.0–6.0	80	0.08	0.110	23
28	PtSn$Al_{0.2}$/SBA-15	0.5	590	55.9–40.5	98.5	0.32	0.104	24
29	Pt-Cu/$MgAl_2O_4$	1	590	25.7–21.0	87.5	0.24	0.011	25
30	Pt-Ag/$MgAl_2O_4$	1	590	30.6–16.7	95.1	0.28	0.034	25
31	Pt-Au/$MgAl_2O_4$	1	590	33.7–16.6	60	0.31	0.040	25
32	Pt-Sn-2/$MgAl_2O_4$	0.55	580	44–18.7	92	0.23	0.014	26
33	Pt-Sn-3/$MgAl_2O_4$	0.53	580	42–18.7	97	0.23	0.013	26
34	Pt-Sn-4/$MgAl_2O_4$	0.5	580	50–31.5	98	0.29	0.009	26
35	Pt-Sn-5/$MgAl_2O_4$	0.42	580	45–37.6	98	0.31	0.003	26
36	Pt-Sn-6/$MgAl_2O_4$	0.39	580	44–38.9	99	0.33	0.002	26
37	Pt/0.5Sn-SBA-15	0.72	580	43.8–38.3	98.5	0.62	0.038	27
38	Pt-Sn/$MgAl_2O_4$-ALT	1	575	33–29	99	0.59	0.057	28
39	$Pt^0Zn^{\delta+}/SiO_2$	3.05	550	30.2–16.1	98.1	0.96	0.027	29
40	$Ga^{\delta+}Pt^0/SiO_2$	1.55	550	31.9–18.2	99	1.36	0.038	3
41		1.55	550	36.5–26.9	90.9	0.76	0.023	3
42		1.55	550	40.7–38.5	63.5	0.04	0.005	3
43	Pt/Mg(Sn)(Al)O	0.5	550	29.4–27.8	93.7	0.64	0.001	10
44	2Pt-0.6Sn/γ–Al_2O_3	2	540	42.9–39.2	93.7	0.09	0.020	30
45	2Pt-1.2Sn/γ–Al_2O_3	2	540	43.5–41.4	96.9	0.09	0.011	30
46	2Pt-2.4Sn/γ–Al_2O_3	2	540	42.8–41.7	98.3	0.09	0.006	30
47	2Pt-3.6Sn/γ–Al_2O_3	2	540	42.8–41.8	98.6	0.09	0.005	30
48	0.1Pt10Cu/Al_2O_3	0.1	520	13.1–12.4	90	0.65	0.001	17
49	Pt-Na-[Fe]/ZSM-5	0.1	520	33–13	98.3	1.51	0.008	31

[a] The first value was obtained at the beginning of the run, and the second at the end. [b]The C_3H_6 selectivity was obtained at the beginning of the run. [c]Defined as (mol reacted propane) per (mol Pt*t(s)). [d]The first-order deactivation model was used to estimate the catalytic stability.[6]

Supplementary Table 4. Summary of the catalytic data of PtGa-Pb and other reported Pt-based catalysts for PDH.

entry	catalyst	Pt (wt%)	temp. (°C)	WHSV (h^{-1})[a]	gas composition	τ (h)[b]	operation time (h)[c]	ref
1	PtIn/Mg(Al)O-600	0.6	620	3.3	$C_3H_8/H_2/Ar = 8/7/35$	37.5	41.0	7
2	PtIn/Mg(Al)O-x	0.6	620	3.3	$C_3H_8/H_2/Ar = 8/7/35$	8.5	8.0	8
3	0.3PtSn/1.5In-Al	0.3	620	3.3	$C_3H_8/H_2/Ar = 8/7/35$	7.0	2.8	9
4	$PtGa/SiO_2$	3	600	30.7	$C_3H_8 = 3.9, H_2 = 5, He = 40$	54.1	50.0	This
5	$PtGa-Pb/SiO_2$ (Pt/Pb = 2)	3	600	30.7	$C_3H_8 = 3.9, H_2 = 5, He = 40$	1159.0	96.0	Study
6	Pt/Mg(Sn)(Al)O	0.5	600	14	$C_3H_8/H_2/Ar = 1/0.5/2$	561.2	48.0	10
7	Pt_3In/SiO_2	0.3	600	3	$C_3H_8/H_2 = 1/1$, balance N_2	57.1	2.5	11
8	Pt3Ga/CeAl	1	600	10	$C_3H_8/H_2 = 1/1$, balance N_2	39.0	15.0	12
9	$Pt3Ga/Al_2O_3$	1	600	10	$C_3H_8/H_2 = 1/1$, balance N_2	29.5	15.0	12
10	Pt/CeAl	1	600	10	$C_3H_8/H_2 = 1/1$, balance N_2	13.2	15.0	12
11	PtSn/TS-1	0.5	600	3	$C_3H_8/H_2/N_2 = 1/1/4$	30.1	7.0	13
12	$15\%Zn-0.1\%/Al_2O_3$	–	600	3	$C_3H_8/H_2 = 1/1$, balance N_2	22.1	4.0	14
13	Pt/TA0	1	600	10	$C_3H_8/H_2/N_2 = 13/13/24$	7.4	10.0	15
14	Pt/TA10	1	600	10	$C_3H_8/H_2/N_2 = 13/13/24$	10.6	10.0	15
15	Pt/TA20	1	600	10	$C_3H_8/H_2/N_2 = 13/13/24$	7.4	10.0	15
16	PtSnIn/08Zr-Al	0.3	600	3.3	$C_3H_8/H_2/Ar = 8/7/35$	10.3	2.5	16
17	$0.1Pt10Cu/Al_2O_3$	0.1	600	4.0	$C_3H_8/H_2 = 1/1$	4.7	4.0	17
18	Pt-Sn/SAPO-34-500	0.5	595	5.6	$C_3H_8/H_2 = 4/1$	6.7	8.0	18
19	Pt/Al_2O_3 sheet	0.36	590	9.4	$C_3H_8/H_2/N_2 = 1:1.25:4$	17.1	24.0	19
20	$PtSn/Al_2O_3$ sheet	0.35	590	9.4	$C_3H_8/H_2/N_2 = 1:1.25:4$	142.6	24.0	19
21	Pt-Sn-Na/Al-SBA-15	0.5	590	3.0	$C_3H_8/H_2 = 75/25$	41.3	40.0	20
22	Pt-Na/Sn-ZSM-5	0.5	590	3.0	$C_3H_8/H_2 = 75/25$	92.6	9.0	21
23	PtNa/Zn(1.0%)-ZSM-5	0.5	590	3.0	$C_3H_8/H_2 = 4/1$	85.1	9.0	22
24	Pt-Sn/mesoporous Al_2O_3	0.5	590	3.0	$C_3H_8/H_2 = 4$	22.8	6.0	23
25	Pt-Sn/ZSM-5	0.5	590	3.0	$C_3H_8/H_2 = 4$	18.4	6.0	23
26	$Pt-Sn/\gamma-Al_2O_3$	0.5	590	3.0	$C_3H_8/H_2 = 4$	17.2	6.0	23
27	Pt-Sn/SBA-15	0.5	590	3.0	$C_3H_8/H_2 = 4$	9.1	6.0	23
28	$PtSnAl_{0.2}/SBA-15$	0.5	590	2.5	$C_3H_8/Ar = 1/5$	9.6	6.0	24
29	$Pt-Cu/MgAl_2O_4$	1	590	6.8	$C_3H_8 = 19, H_2 = 19, He = 2$	89.2	24.0	25
30	$Pt-Ag/MgAl_2O_4$	1	590	6.8	$C_3H_8 = 19, H_2 = 19, He = 2$	29.8	24.0	25
31	$Pt-Au/MgAl_2O_4$	1	590	6.8	$C_3H_8 = 19, H_2 = 19, He = 2$	25.1	24.0	25
32	$Pt-Sn-2/MgAl_2O_4$	0.55	580	2.4	$C_3H_8 = 2, H_2 = 2, He = 16$	73.3	90.0	26
33	$Pt-Sn-3/MgAl_2O_4$	0.53	580	2.4	$C_3H_8 = 2, H_2 = 2, He = 16$	78.5	90.0	26
34	$Pt-Sn-4/MgAl_2O_4$	0.5	580	2.4	$C_3H_8 = 2, H_2 = 2, He = 16$	115.9	90.0	26
35	$Pt-Sn-5/MgAl_2O_4$	0.42	580	2.4	$C_3H_8 = 2, H_2 = 2, He = 16$	294.2	90.0	26
36	$Pt-Sn-6/MgAl_2O_4$	0.39	580	2.4	$C_3H_8 = 2, H_2 = 2, He = 16$	427.8	90.0	26
37	Pt/0.5Sn-SBA-15	0.72	580	8.3	$C_3H_8/Ar = 7/3$	26.4	6.0	27
38	$Pt-Sn/MgAl_2O_4-ALT$	1	575	14.7	$C_3H_8/H_2 = 1.2$, balance He	17.6	3.3	28
39	$Pt^0Zn^{\delta+}/SiO_2$	3.05	550	75	$C_3H_8/Ar = 10/40$	36.8	3.5	29
40	$Ga^{\delta+}Pt^0/SiO_2$	1.55	550	98.3	$C_3H_8/Ar = 1:4$	26.5	20.0	3
41		1.55	550	43.3	$C_3H_8/Ar = 1:4$	44.2	20.0	3
42		1.55	550	2.0	$C_3H_8/Ar = 1:4$	214.1	20.0	3
43	Pt/Mg(Sn)(Al)O	0.5	550	14	$C_3H_8/H_2/Ar = 1/0.5/2$	1531.2	240.0	10
44	$2Pt-0.6Sn/\gamma-Al_2O_3$	2	540	3.5	$C_3H_8/H_2/N_2 = 6/2/42$	49.0	6.0	30
45	$2Pt-1.2Sn/\gamma-Al_2O_3$	2	540	3.5	$C_3H_8/H_2/N_2 = 6/2/42$	87.2	6.0	30
46	$2Pt-2.4Sn/\gamma-Al_2O_3$	2	540	3.5	$C_3H_8/H_2/N_2 = 6/2/42$	166.4	6.0	30
47	$2Pt-3.6Sn/\gamma-Al_2O_3$	2	540	3.5	$C_3H_8/H_2/N_2 = 6/2/42$	183.0	6.0	30
48	$0.1Pt10Cu/Al_2O_3$	0.1	520	4.0	$C_3H_8/H_2 = 1/1$	1906.6	120.0	17
49	Pt-Na-[Fe]/ZSM-5	0.1	520	15.1	$C_3H_8/He = 25/75$	125.0	166.0	31

[a] WHSV: weight hourly space velocity (h^{-1}). [b] Expected catalyst life defined as $\tau = k_d^{-1}$. [c] operation time: total time tested for a single run.

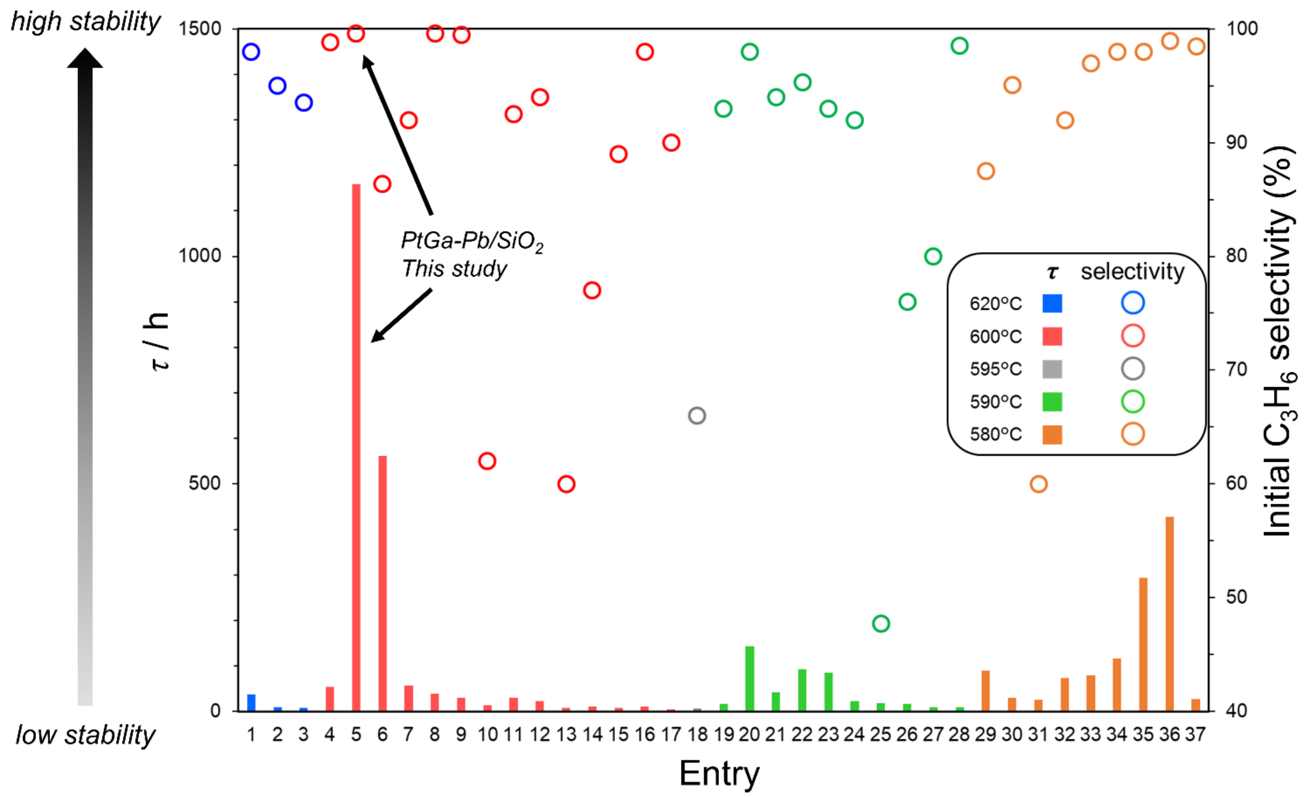

Supplementary Figure 11. At-a-glance chart of the catalytic performance of PtGa-Pb/SiO_2 (Pt/Pb = 2) and existing Pt-based catalysts in PDH (references are listed in Supplementary Tables 3 and 4). Expected catalyst life ($\tau = k_d^{-1}$) and initial C_3H_6 selectivity are categorized by reaction temperature (580~620°C). The results of previous studies conducted at temperatures lower than 570°C and higher than 630°C were omitted due to low C_3H_8 conversion or production rate and the lack of long-term stability, respectively.

Supplementary Table 5. Summary of catalytic performance of SiO_2-supported Pt-based materials in PDH at 600°C.

Catalyst	C_3H_8 Conversion (%) at 0.5 h	C_3H_8 Conversion (%) at 50 h	k_d (h^{-1})[a]	τ (h)[b]	C_3H_6 selectivity (%) at 0.5 h
Pt	30.7	10.8	0.026	38.2	86.8
Pt-Ga (Pt/Ga = 3)	38.9	17.7	0.022	45.6	89.8
PtGa	44.7	24.5	0.018	54.1	98.8
PtGa-Pb (Pt/Pb = 2)	30.3 (4 h)	28.4 (96 h)	0.001	1159	99.6
Pt_3Sn	39.1	18.0	0.022	46.1	97.5
PtSn	33.6	21.0	0.013	76.9	99.2
Pt_3In	34.6	13.9	0.024	41.7	94.9
Pt-Pb (Pt/Pb = 3)	28.8	13.7	0.019	53.0	96.1

[a] Deactivation rate constant. [b] Expected catalyst life.

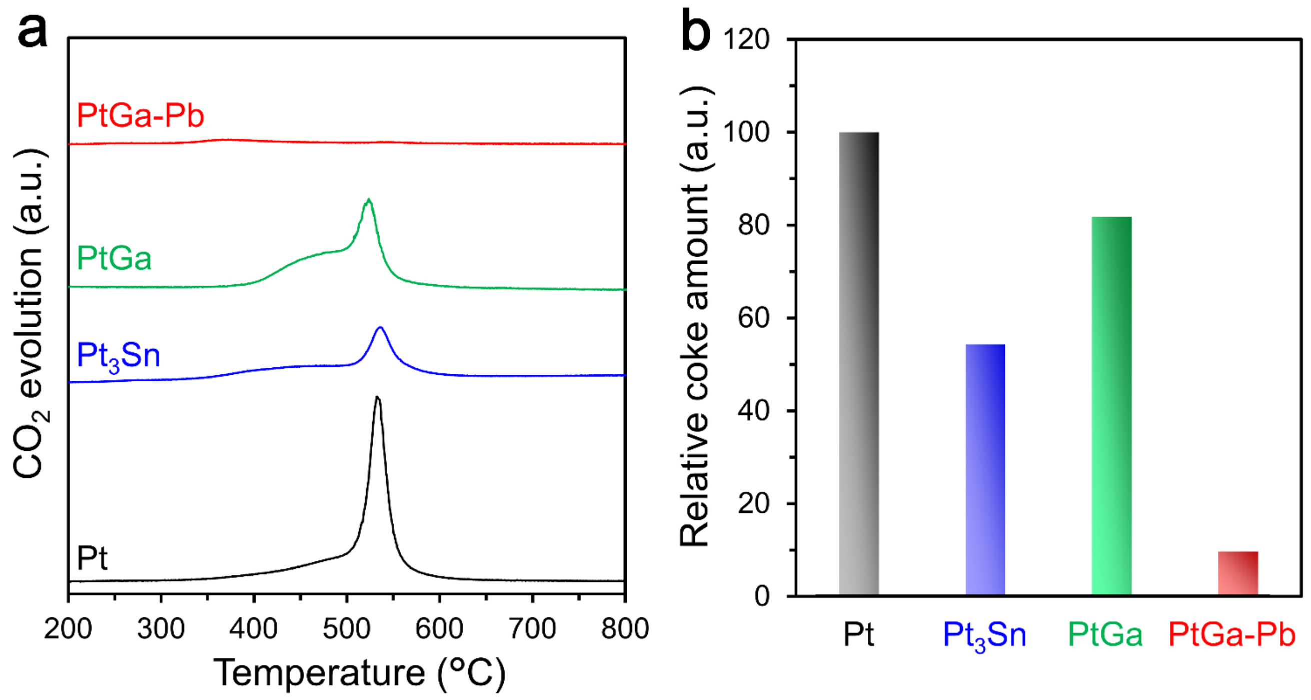

Supplementary Figure 12. a Temperature-programmed oxidation (TPO) profiles of the spent catalysts (without quartz sand) in the dehydrogenation of propane at 600°C for 20 h. **b** The relative coke amount accumulated on the catalysts (200–800°C) estimated from TPO experiment.

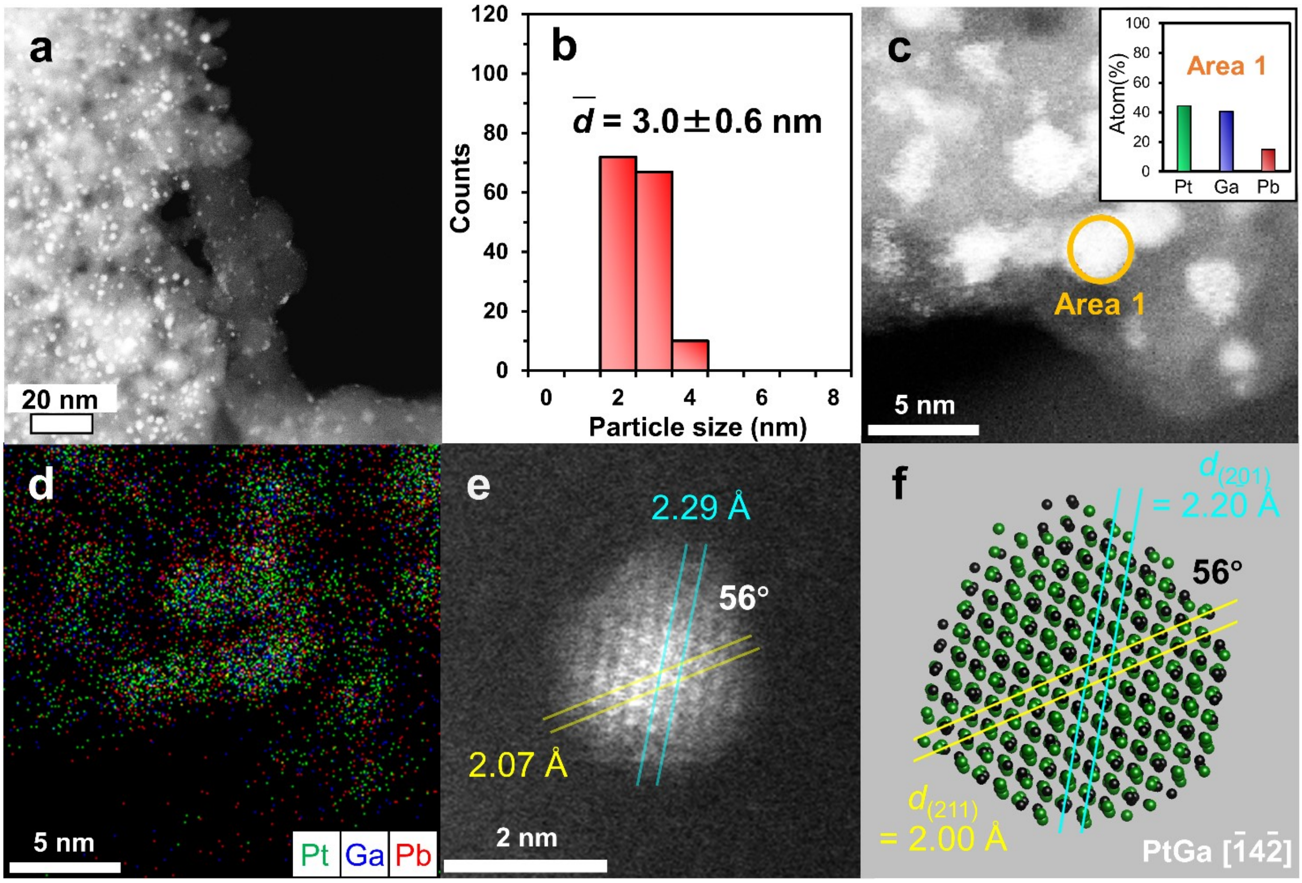

Supplementary Figure 13. a HAADF-STEM image of the spent PtGa-Pb/SiO_2 (Pt/Pb = 2) in PDH (600°C for 50 h) and **b** the size distribution of nanoparticles. The particle size distribution was narrow with small and uniform nanoparticles. The particle sizes ranged from 1 nm to 4 nm with a volume weighted average of 3.0 ± 0.6 nm. The aggregation of the nanoparticles was not observed before (2.8 ± 0.6 nm) and after (3.0 ± 0.6 nm) the dehydrogenation of propane at 600°C for 50 h. **c** HAADF-STEM image of the spent PtGa-Pb/SiO_2 (Pt/Pb = 2) and **d** corresponding elemental map of Pt, Ga, Pb acquired by EDS. Inset in **c** shows the atom% included in area 1 (orange circle). **e** HAADF-STEM image of a single nanoparticle in the spent PtGa-Pb/SiO_2 (Pt/Pb = 2). **f** Crystal structure of intermetallic PtGa viewed along $[\bar{1}4\bar{2}]$ direction.

Supplementary Note 5. Characterization of the spent PtGa-Pb/SiO_2: Supplementary Figures 13a and b show the HAADF-STEM image of spent PtGa-Pb/SiO_2 (Pt/Pb = 2) and the particle size distribution, respectively. The particle size distribution was narrow (mostly 1.5~3 nm) with a volume weighted average of 3.0 ± 0.6 nm, which is consistent with that of the flesh PtGa-Pb/SiO_2 (Pt/Pb) (Supplementary Figure 1), indicating the high resistance against the nanoparticle sintering. Supplementary Figures 13c and d shows the HAADF-STEM image of the spent PtGa-Pb/SiO_2 (Pt/Pb = 2) and corresponding elemental map acquired by EDX analysis, respectively. The atom% in the whole nanoparticle was consistent with that of the flesh PtGa-Pb/SiO_2 (Pt/Pb = 2) (Fig. 2). Supplementary Figure 13e shows the high-resolution HAADF-STEM image of the spent PtGa-Pb/SiO_2 (Pt/Pb = 2) with a single nanoparticle. A crystal structure with interplanar distances of 2.07 Å and 2.29 Å and the dihedral angles of 56° were observed, which agree with those of (211) and $(20\bar{1})$ planes of intermetallic PtGa viewed along with $[\bar{1}4\bar{2}]$ direction (Supplementary Figure 13f).

Supplementary Note 6. PtGa-M/SiO_2 (M = Pb, Sn, and In; Pt/M = 2): we also tested different third metal elements for the modification of PtGa (PtGa-M: M = Pb, Sn, and In: Pt/M = 2: see Supplementary Table 2 for Pt dispersion). These trimetallic catalysts were also prepared in a similar method used for PtGa-Pb/SiO_2 and tested in PDH at 650°C (Supplementary Figure 14). Only PtGa-Pb/SiO_2 exhibited a drastic improvement in the catalytic performance, while the other catalysts did not. This results demonstrated that Pb acted as an effective promoter for PtGa. This propane dehydrogenation is typically performed at 550~600°C to avoid the sintering of nanoparticles and coke formation, which originated from side reactions. Here, we employed a relatively harsh condition to obtain the deactivation trend and C_3H_6 selectivity change trend in a short period of days.

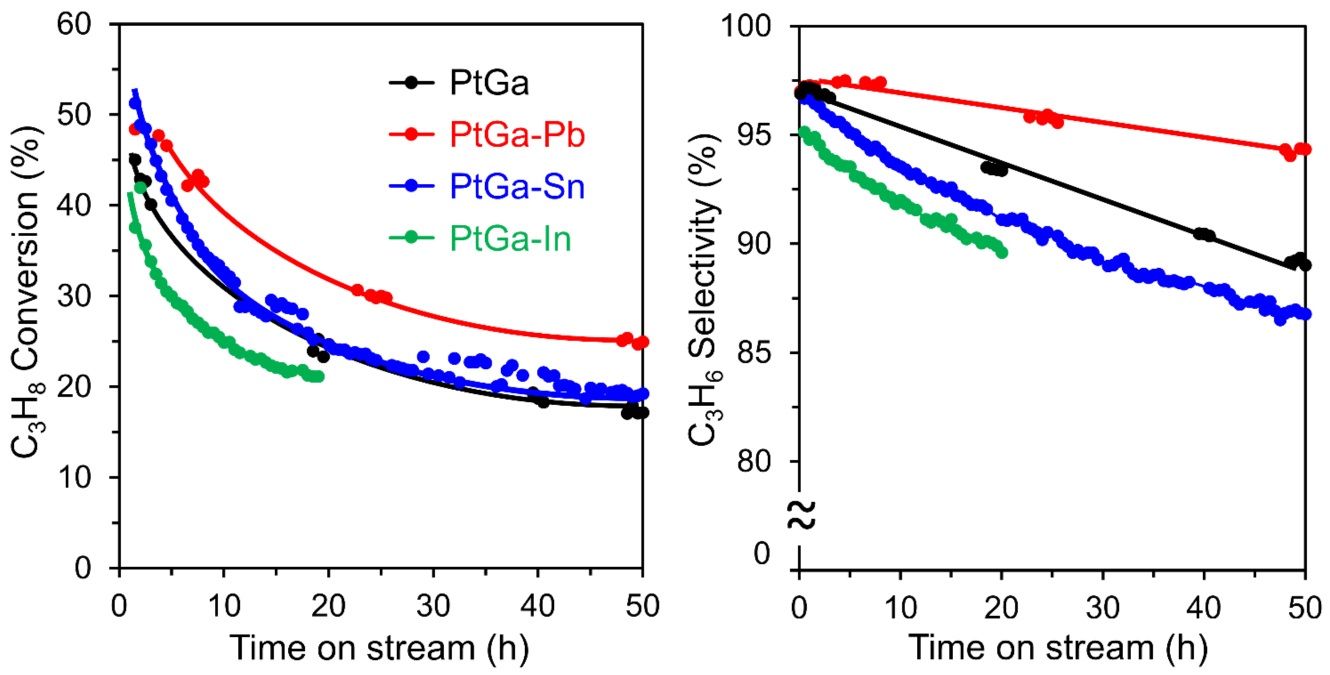

Supplementary Figure 14. Catalytic performances of PtGa-M/SiO_2 (M = Pb, Sn, and In: Pt/M = 2) in PDH at 650°C.

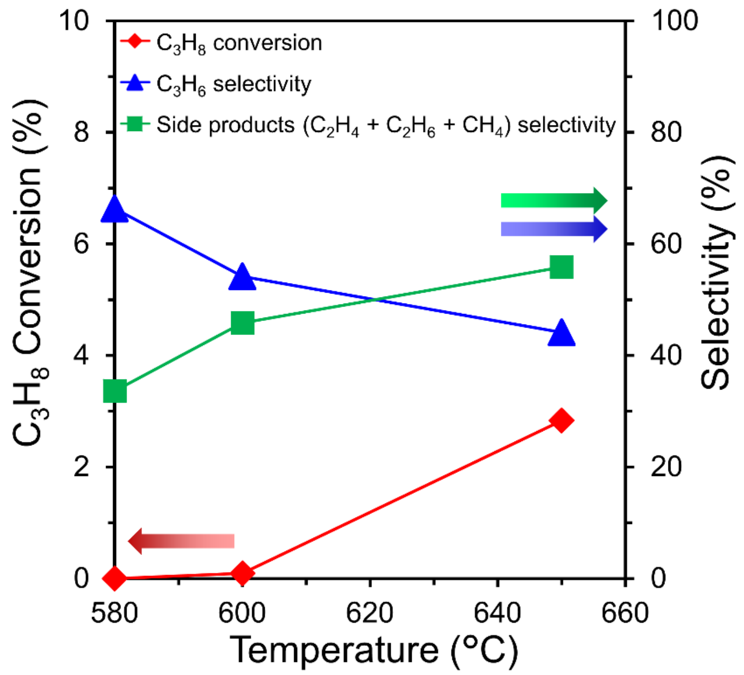

Supplementary Figure 15. Control experiment in PDH without catalyst (only with diluent quartz sand) at 580, 600, and 650°C. Fragmented C_1 and C_2 were formed by thermal cracking of propane particularly at 650°C, while very limited at 580, 600°C and the lower.

Supplementary Note 7. Silica-supported Pt-based catalysts: a series of silica-supported Pt-based bimetallic catalysts (Pt_xM/SiO_2, where x = 3, and M = Ga, Sn, In, and Pb, and PtSn) were prepared by the co-impregnation method using freeze-drying. To identify the resulting bimetallic phases, obtained catalysts were analyzed by powder XRD (Supplementary Figure 16). The Pt dispersions estimated by CO pulse chemisorption are listed in Supplementary Table 2. $PtSn/SiO_2$ likely has Pt_3Sn-PtSn core-shell structure considering the XRD pattern and Pt dispersion lower than that of Pt_3Sn. This is consistent with the fact that alloying with Sn proceeds from outside of Pt nanoparticles, which can be formed at the early stage of H_2 reduction. Therefore, PtSn phase is expected to work as the main active site.

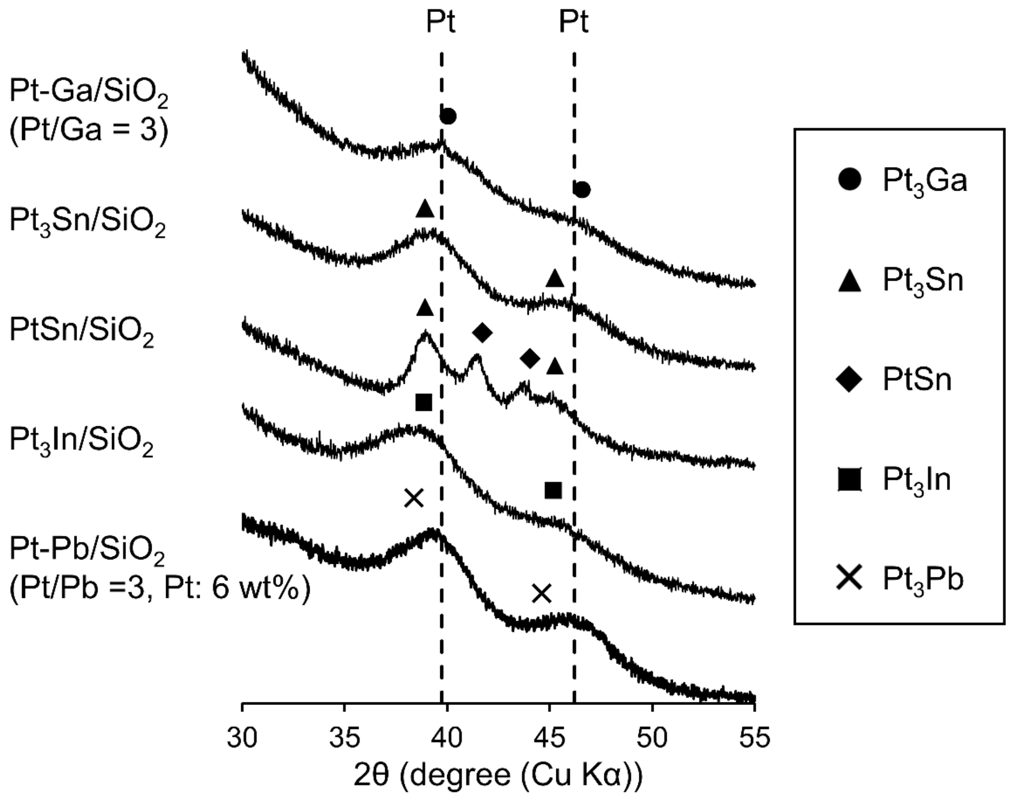

Supplementary Figure 16. X-ray diffraction (XRD) patterns of the silica-supported Pt-based bimetallic catalysts (Pt: 3 wt%). Pt-Pb/SiO_2 (Pt/Pb = 3) with 6 wt% Pt loading was used for better visibility. Black dotted lines indicate the diffraction angles of pure Pt. The desired intermetallic phase was observed for each sample. References: Pt,[32] Pt_3Ga,[33] Pt_3Sn,[34] PtSn,[34] Pt_3In.[35], and Pt_3Pb.[36]

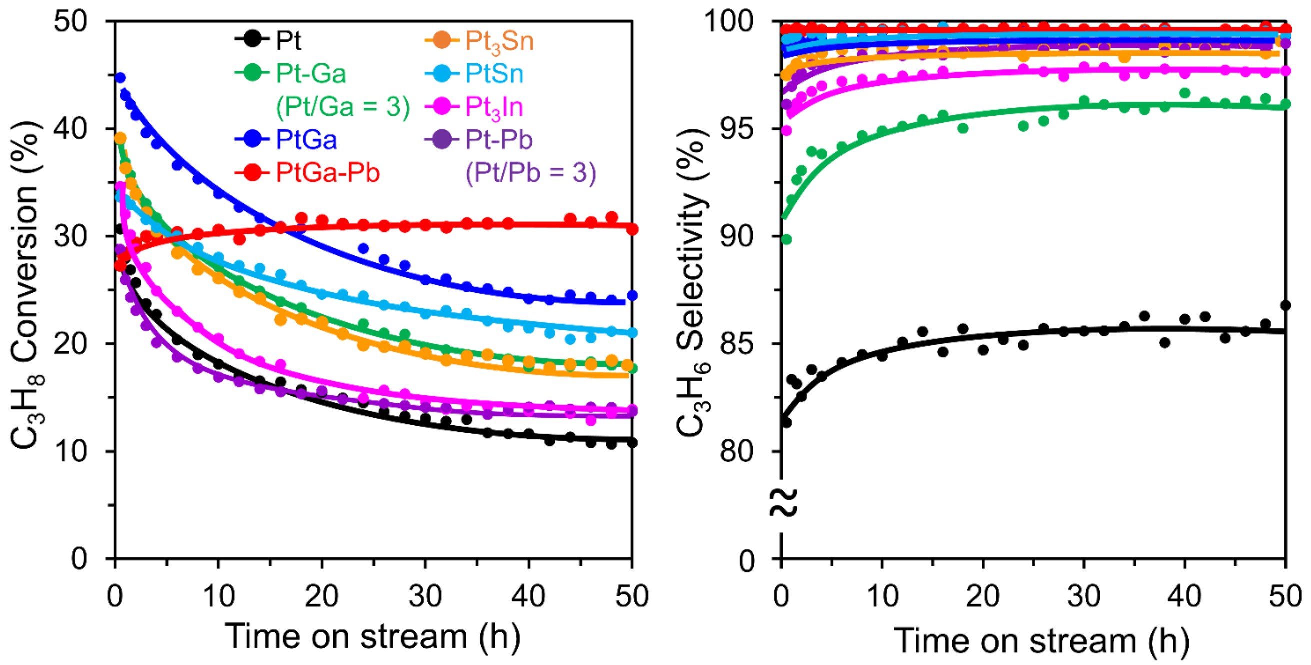

Supplementary Figure 17. Propane dehydrogenation on silica-supported Pt-based catalysts at 600°C.

Supplementary Note 8. Regeneration process: we tested the recyclability of the prepared catalyst. Prior to the second catalytic run, the spent catalysts were calcined under flowing O_2/He (5% for 5 h or 20% for 1 h) at 550°C and subsequently reduced under flowing H_2 at 650°C for 0.5 h. We chose the calcination temperature of 550°C according to literature of a Pt–Ga system, where Pt dispersion drastically decreased after oxidation at 650°C, whereas it retained at 550°C.[37] This result indicates that oxidation at 650°C results in some irreversible structural changes (probably, severe oxidative dealloying or sintering). Considering our TPO experiments shown in Supplementary Figure 12, the coke accumulated on the spent catalysts was completely combusted at 550°C, demonstrating that the oxidation temperature of 550°C is high enough for coke removal. Supplementary Figure 18a shows the changes of catalytic performance of PtGa-Pb/SiO_2 catalyst before and after a regeneration process with 5% O_2. Upon the regeneration, the catalytic performance was recovered to the original level after a short induction period. This induction period might be attributed to catalyst reconstruction: the catalyst structure (possibly, the placement of Pb atoms at the surface of PtGa) may be partly rebuilt during the regeneration process and further reconstruction to the original state occurs during the initial state of the 2nd catalytic run. We also tested the different calcination condition under 20% O_2/He (as a model for aerobic oxidation) for PtGa-Pb, which showed a similar trend with a longer induction period (Supplementary Figure 18b). Thus, the catalyst can be reused by the simple regeneration procedure and has high resistance against the sintering of nanoparticles, as reported for Pt–Ga bimetallic systems.[38,39] The sintering of nanoparticles is one of the biggest issues in the dehydrogenation of propane.[6] On the other hand, other Sn-containing bimetallic (Pt_3Sn and PtSn) and trimetallic (PtGa-Sn) catalysts did not recovered the original catalytic performances after the regeneration process (Supplementary Figure 19). Particularly for PtSn, the C_3H_8 conversion and C_3H_6 selectivity significantly dropped. Thus, the PtGa-Pb/SiO_2 catalyst has a better recyclability than the Sn-containing catalysts.

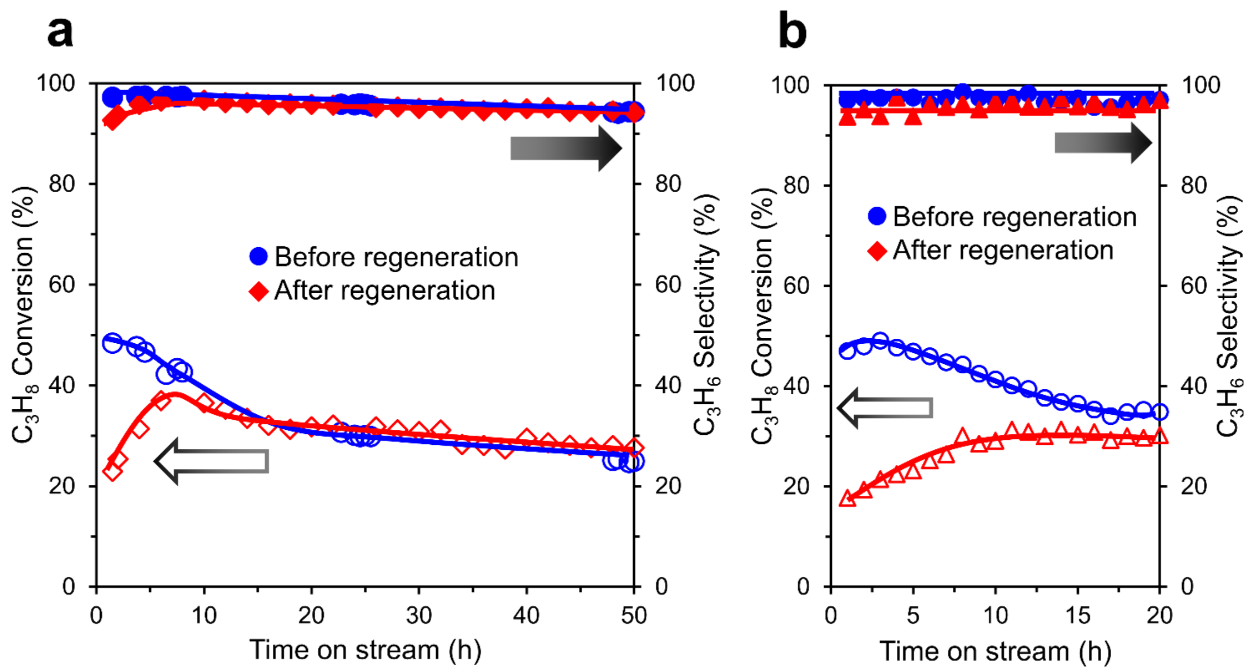

Supplementary Figure 18. Catalytic performances of PtGa-Pb/SiO_2 (Pt/Pb = 2) at 650°C before (first run) and after (second run) regeneration. The regeneration process was carried out under flowing **a** 5% O_2/He (F = 50 mL min^{-1}) at 550°C for 5 h and **b** 20% O_2/He (F = 50 mL min^{-1}) at 550°C for 1 h, and the subsequent H_2 reduction at 650°C for 0.5 h.

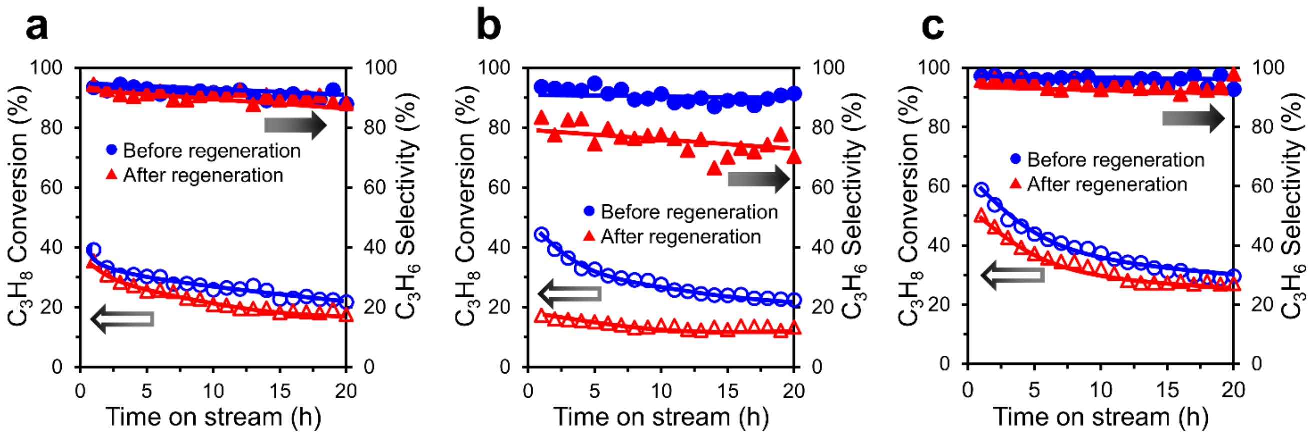

Supplementary Figure 19. Effect of regeneration process for the dehydrogenation of propane on **a** Pt_3Sn/SiO_2, **b** $PtSn/SiO_2$, and **c** $PtGa\text{-}Sn/SiO_2$ (Pt/Sn = 2) catalysts at 650°C. The regeneration process was carried out under flowing 20% O_2/He (F = 50 mL min^{-1}) at 550°C for 1 h and the subsequent H_2 reduction at 650°C for 0.5 h.

Supplementary Note 9. Amount of additive Pb on $PtGa/SiO_2$: a series of $PtGa\text{-}Pb/SiO_2$ (Pt/Pb = 5, 2.5, 2, and 1.5) catalysts was tested in PDH at 650°C (Supplementary Figure 20, see Supplementary Table 2 for the dispersions of Pt). The catalytic performance was enhanced when the Pb content increased up to Pt/Pb = 2. Further increase in Pb content (Pt/Pb = 1.5) results in a drop of the catalytic performance particularly at the early stage of the reaction. Interestingly, the catalytic activity of $PtGa\text{-}Pb/SiO_2$ (Pt/Pb = 1.5) was recovered to that of $PtGa\text{-}Pb/SiO_2$ (Pt/Pb = 2) after a short induction period. This implies that the overloaded Pb atoms excessively block the active Pt sites (actually, Pt dispersion decreased, Supplementary Table 2), while spill-overed to release the Pt sites during the catalytic run. According to these results, we concluded that the Pt/Pb ratio of 2 was the best for our catalytic system.

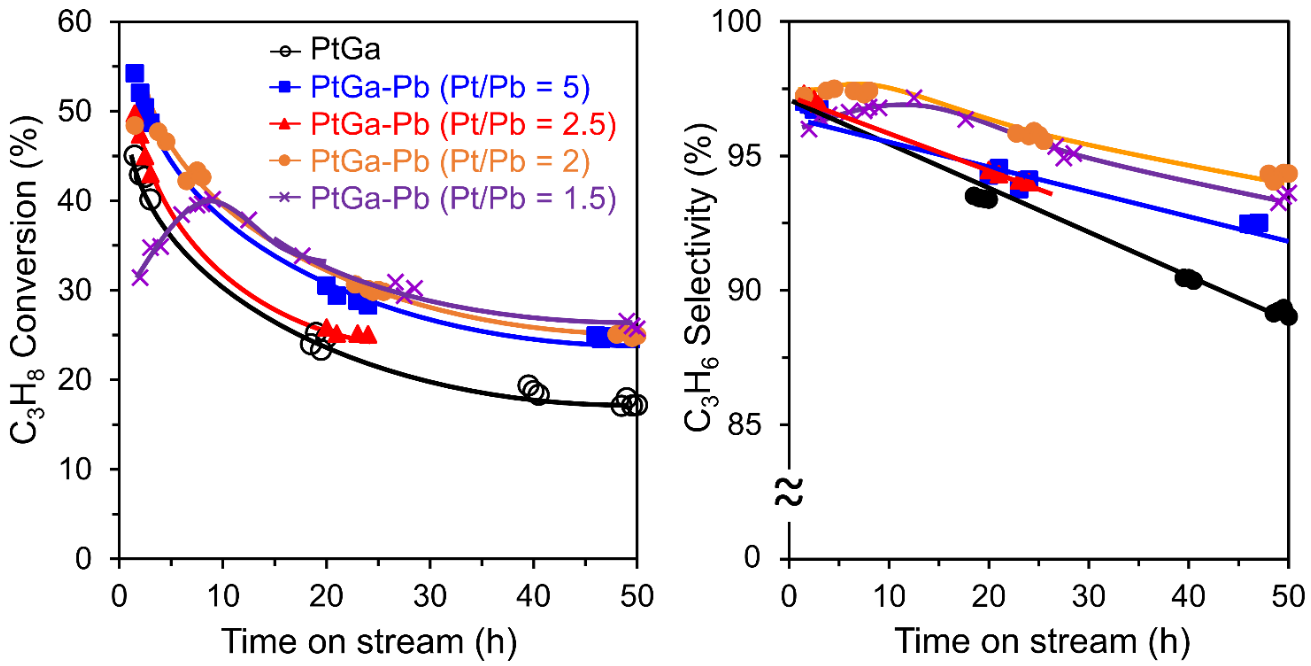

Supplementary Figure 20. Catalytic performances of $PtGa/SiO_2$ and $PtGa\text{-}Pb/SiO_2$ (Pt/Pb = 5, 2.5, 2, and 1.5) in PDH at 650°C.

Supplementary Note 10. Effect of Support for PtGa bimetallic catalyst: we surveyed catalyst supports for PtGa. In this study, PtGa supported on a series of oxide supports (Al_2O_3, $MgAl_2O_4$, CeO_2, $CeZrO_2$, ZrO_2, $CaZrO_3$, and TiO_2) were prepared by the conventional impregnation method with an excess amount of water and tested for the dehydrogenation of propane. Supplementary Figure 21 shows the time on stream of C_3H_6 yield and C_3H_6 selectivity during the dehydrogenation of propane on PtGa bimetallic catalyst supported on the various oxide supports. In general, Al_2O_3[40,41] and $MgAl_2O_4$[42] supports have been used for propane dehydrogenation owing to the hydrothermal stability and capability of dispersing small Pt nanoparticles by the presence of Al cations. These supports showed comparatively high activity and selectivity compared to the other oxide supports (except for SiO_2). Notably, PtGa/SiO_2 (prepared by the co-impregnation method using freeze-drying) exhibited the highest activity and selectivity in this catalyst support survey.

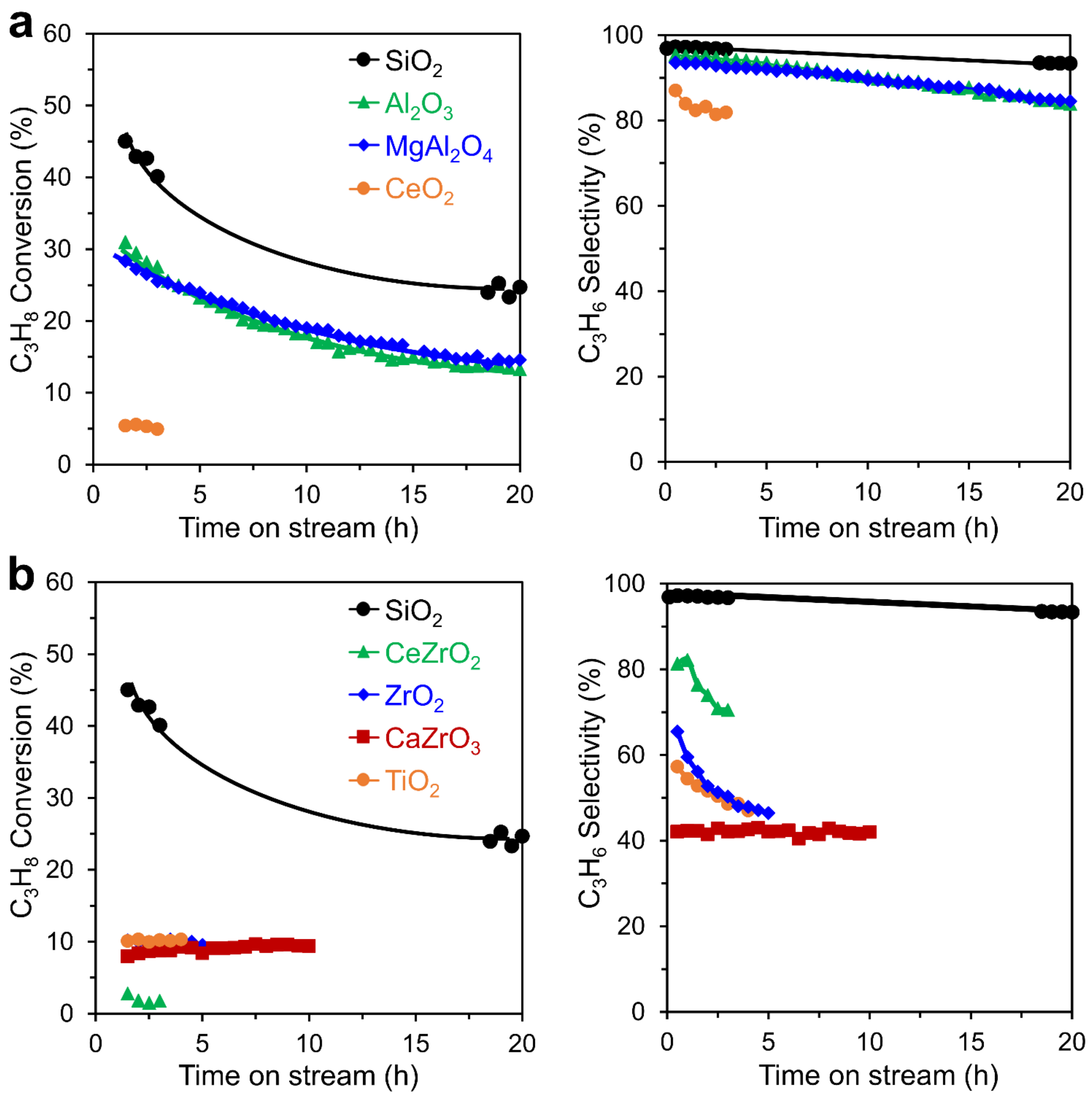

Supplementary Figure 21. a, b Catalytic performances of PtGa supported on various oxides in PDH at 650°C.

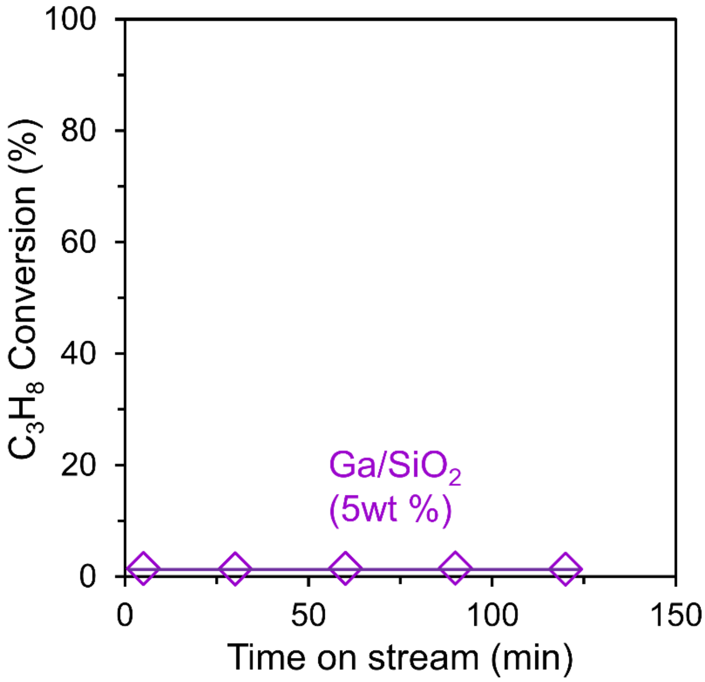

Supplementary Figure 22. Catalytic performances of Ga/SiO_2 (Ga: 5 wt%) in PDH at 650°C.

Pt_3Sn-Pt_3 site

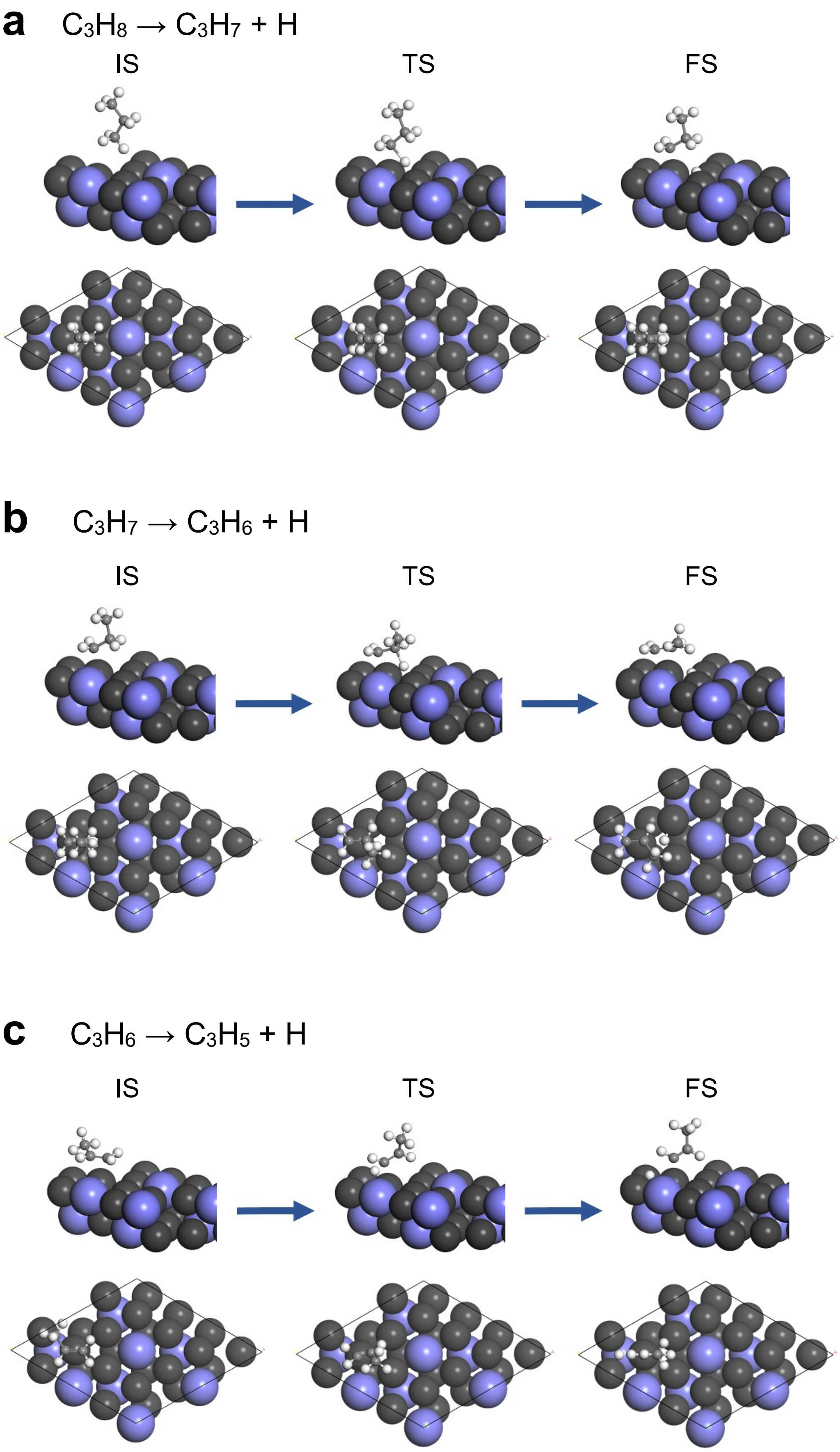

Supplementary Figure 23. Structures of initial (IS), transition (TS), and final states (FS) of **a** 1st, **b** 2nd, and **c** 3rd C–H scissions in PDH on the $Pt_3Sn(111)$-Pt_3 site.

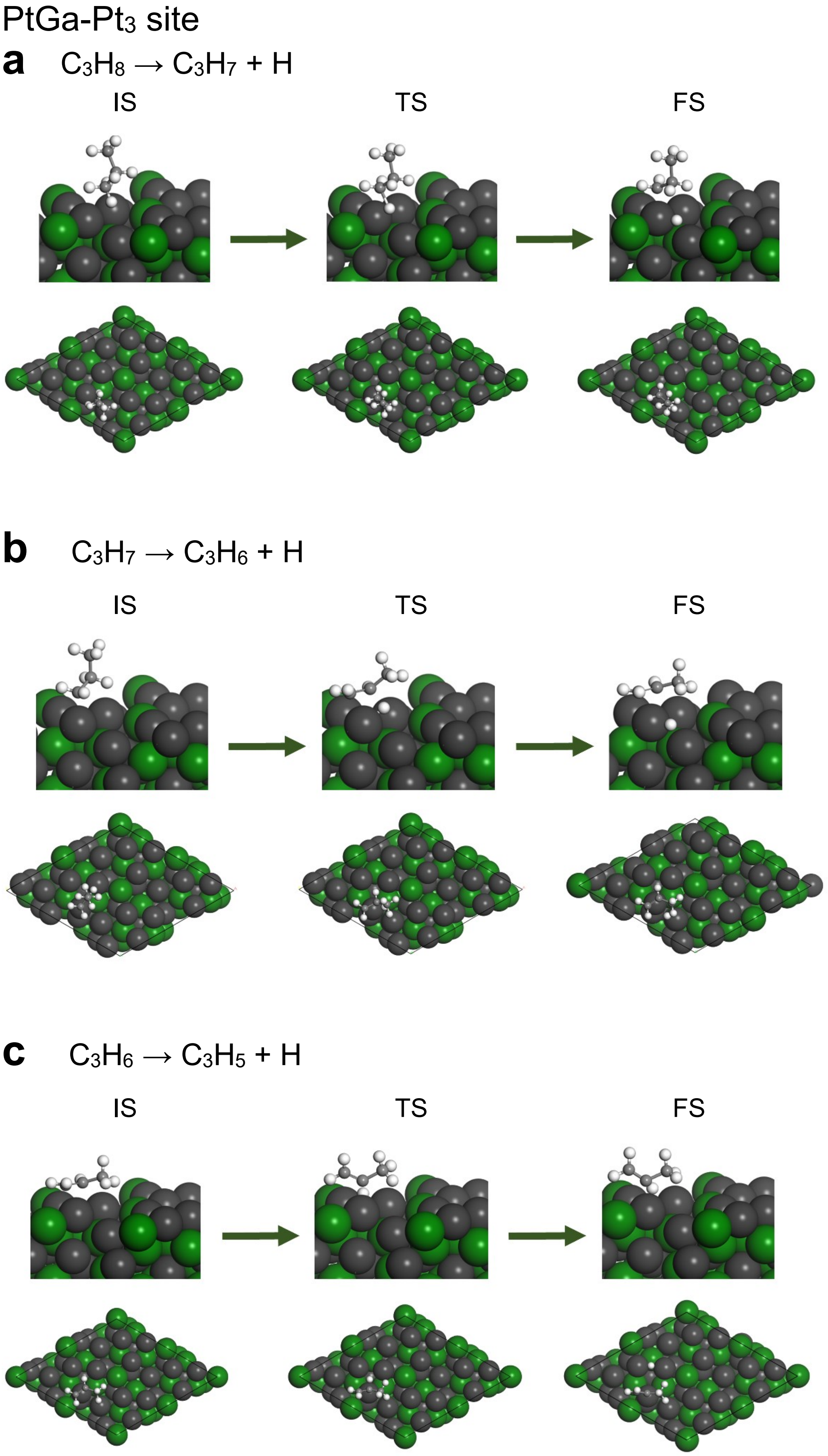

Supplementary Figure 24. Structures of initial (IS), transition (TS), and final states (FS) of **a** 1st, **b** 2nd, and **c** 3rd C–H scissions in PDH on the PtGa:A(111)-Pt_3 site.

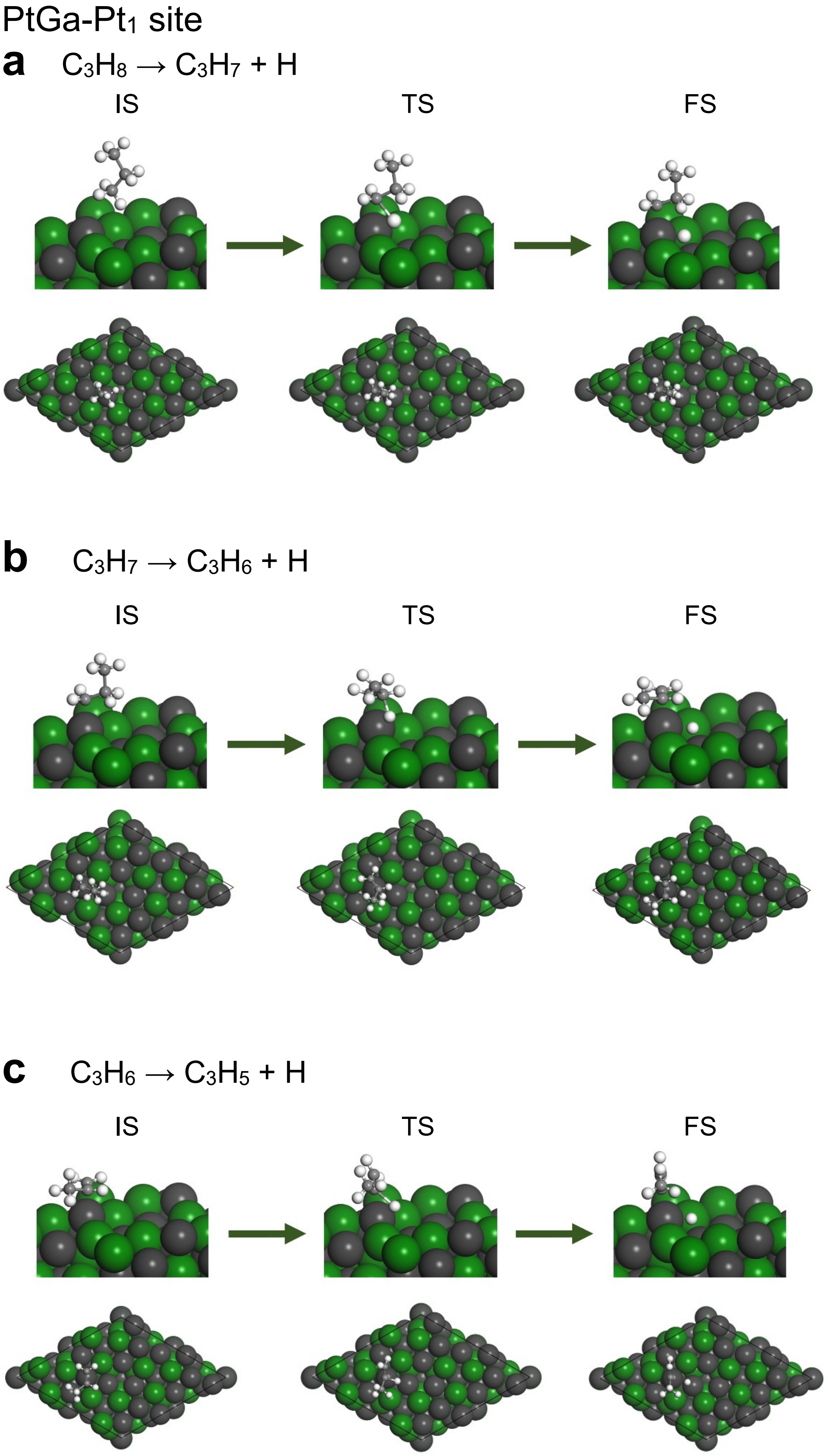

Supplementary Figure 25. Structures of initial (IS), transition (TS), and final states (FS) of **a** 1st, **b** 2nd, and **c** 3rd C–H scissions in PDH on the PtGa:A(111)-Pt$_1$ site.

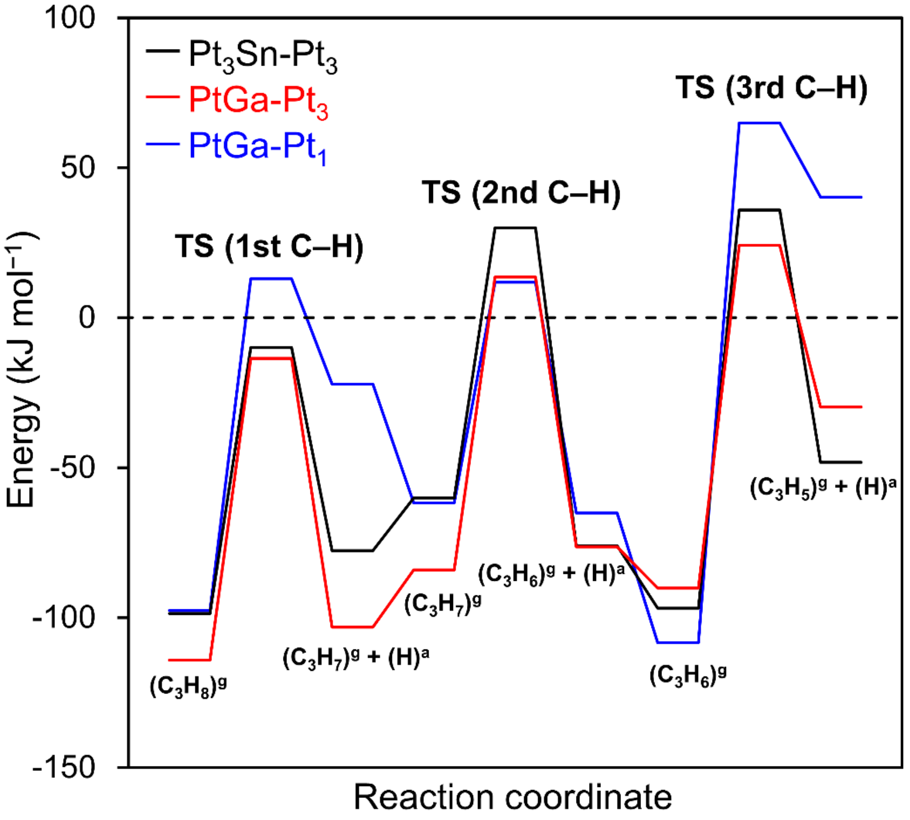

Supplementary Figure 26. Energy diagram for PDH on the PtGa:A(111)-Pt_1, PtGa:A(111)-Pt_3, and Pt_3Sn(111)-Pt_3 sites calculated by density functional theory (DFT). The superscript "g" and "a" indicate the gaseous and adsorbed states of molecules, respectively.

Supplementary Table 6. Calculated activation energy (E_a) for the dehydrogenation of propane on Pt-based surfaces.

	Activation energy (kJ mol^{-1})			
Surface	1st C–H scission ($E_a^{\text{1st C–H}}$)[a]	2nd C–H scission ($E_a^{\text{2nd C–H}}$)[b]	C_3H_6 desorption (E_a^{des})[c]	3rd C–H scission ($E_a^{\text{3rd C–H}}$)[d]
Pt_3Sn-Pt_3	100.5	97.6	90.1	114.3
PtGa-Pt_3	88.7	90.0	96.8	132.7
PtGa-Pt_1	110.6	73.6	108.3	173.2

[a] Activation energy for first C–H scission ($C_3H_8 \rightarrow C_3H_7 + H$). [b] Activation energy for second C–H scission ($C_3H_7 \rightarrow C_3H_6 + H$). [c] Activation energy for third C–H scission ($C_3H_6 \rightarrow C_3H_5 + H$). [d] Activation energy for C_3H_6 desorption.

Supplementary Note 11. Estimation of C_3H_6 selectivity on the basis of Arrhenius equation:

$$R = \frac{r_{des}}{r_{3rd}} = \frac{k_{des}\theta_{C_3H_6}}{k_{3rd}\theta_{C_3H_6}(1-\theta)} \approx \frac{v_{des}\exp\left(-\frac{\Delta E_{des}}{RT}\right)}{v_{3rd}\exp\left(-\frac{\Delta E_{3rd}}{RT}\right)} = \frac{v_{des}}{v_{3rd}}\exp\left(\frac{\Delta E}{RT}\right) \approx \exp\left(\frac{\Delta E}{RT}\right) \quad (3)$$

$$C_3H_6\text{selectivity }(\%) = \frac{r_{des}}{r_{3rd} + r_{des}} \times 100 = \frac{R}{1+R} \times 100 \quad (4)$$

Where, r_x, k_x, θ_x, $1-\theta$, and v_x are reaction rate, rate constant, coverage, vacant site fraction, and preexponential factor, respectively. When C_3H_8 conversion is sufficiently low, $1-\theta$ can be approximated as 1. In this estimation, the two preexponential factors were considered nearly equal.

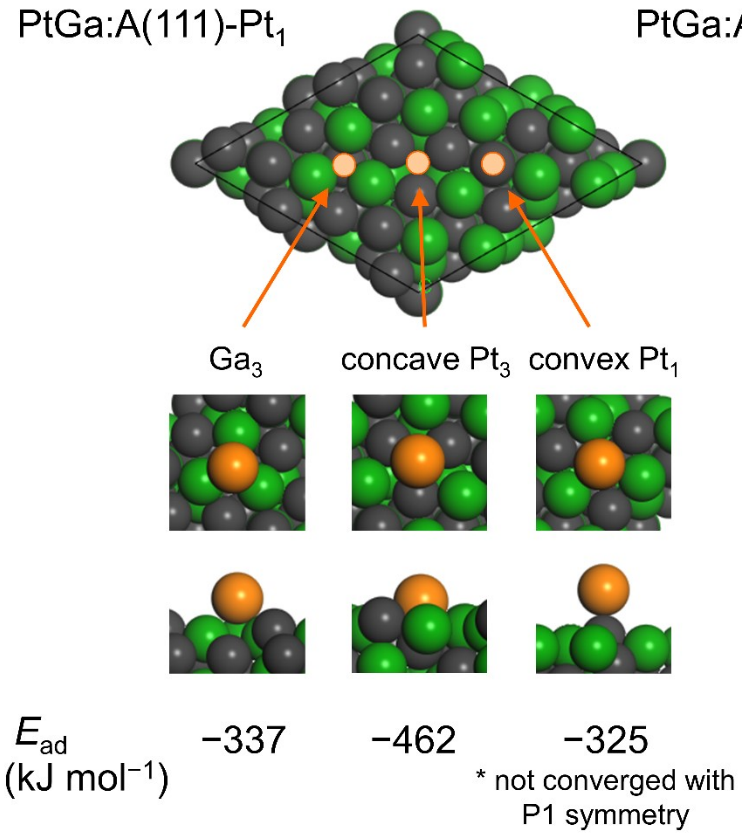

Supplementary Figure 27. Adsorption energy (E_{ad}) and optimized structure of a Pb atom on various Ga_3, Pt_3, and Pt_1 sites of the two PtGa:A(111) surfaces. All calculations were done with P3 symmetry. Structure optimization for Pb on the convex Pt_1 site did not converged with P1 symmetry due to the complete migration of the Pb atom downward, which indicates that the convex Pt_1 site is actually unfavorable for Pb deposition.

Supplementary References

1. Hsu, L. S., Guo, G. Y., Denlinger, J. D. & Allen, J. W. Experimental and theoretical study of the electronic structure of $PtGa_2$. *Phys. Rev. B: Condens. Matter Mater. Phys.* **63**, 1551051–1551058 (2001).
2. Iihama, S., Furukawa, S. & Komatsu, T. Efficient Catalytic System for Chemoselective Hydrogenation of Halonitrobenzene to Haloaniline Using PtZn Intermetallic Compound. *ACS Catal.* **6**, 742–746 (2016).
3. Searles, K. *et al.* Highly Productive Propane Dehydrogenation Catalyst Using Silica-Supported Ga-Pt Nanoparticles Generated from Single-Sites. *J. Am. Chem. Soc.* **140**, 11674–11679 (2018).
4. Wowsnick, G. *et al.* Surface dynamics of the intermetallic catalyst Pd_2Ga, Part II - Reactivity and stability in liquid-phase hydrogenation of phenylacetylene. *J. Catal.* **309**, 221–230 (2014).
5. Wowsnick, G. *et al.* Surface dynamics of the intermetallic catalyst Pd_2Ga, Part i - Structural stability in UHV and different gas atmospheres. *J. Catal.* **309**, 209–220 (2014).
6. Sattler, J. J. H. B., Ruiz-Martinez, J., Santillan-Jimenez, E. & Weckhuysen, B. M. Catalytic dehydrogenation of light alkanes on metals and metal oxides. *Chem. Rev.* **114**, 10613–10653 (2014).
7. Shen, L. L. *et al.* The effects of calcination temperature of support on PtIn/Mg(Al)O catalysts for propane dehydrogenation reaction. *Chem. Eng. J.* **324**, 336–346 (2017).
8. Li, P. P. *et al.* The promotion effects of Ni on the properties of Cr/Al catalysts for propane dehydrogenation reaction. *Appl. Catal. A: Gen.* **522**, 172–179 (2016).
9. Liu, X. *et al.* Improved catalytic performance in propane dehydrogenation of PtSn/γ-Al_2O_3 catalysts by doping indium. *Chem. Eng. J.* **247**, 183–192 (2014).
10. Zhu, Y. *et al.* Lattice-confined Sn (IV/II) stabilizing raft-like Pt clusters: High selectivity and durability in propane dehydrogenation. *ACS Catal.* **7**, 6973–6978 (2017).
11. Zha, S. *et al.* Identification of Pt-based catalysts for propane dehydrogenation: Via a probability analysis. *Chem. Sci.* **9**, 3925–3931 (2018).
12. Wang, T. *et al.* Effects of Ga doping on Pt/CeO_2-Al_2O_3 catalysts for propane dehydrogenation. *AIChE J.* **62**, 4365–4376 (2016).
13. Li, J. *et al.* Size effect of TS-1 supports on the catalytic performance of PtSn/TS-1 catalysts for propane dehydrogenation. *J. Catal.* **352**, 361–370 (2017).
14. Liu, G. *et al.* Platinum-Modified ZnO/Al_2O_3 for Propane Dehydrogenation: Minimized Platinum Usage and Improved Catalytic Stability. *ACS Catal.* **6**, 2158–2162 (2016).
15. Jiang, F. *et al.* Propane Dehydrogenation over Pt/TiO_2-Al_2O_3 Catalysts. *ACS Catal.* **5**, 438–447 (2015).
16. Long, L. L. *et al.* The comparison and optimization of zirconia, alumina, and zirconia-alumina supported PtSnIn trimetallic catalysts for propane dehydrogenation reaction. *J. Ind. Eng. Chem.* **51**, 271–280 (2017).
17. Sun, G. *et al.* Breaking the scaling relationship via thermally stable Pt/Cu single atom alloys for catalytic dehydrogenation. *Nat. Commun.* **9**, 4454 (2018).
18. Nawaz, Z., Tang, X., Chu, Y. & Wei, F. Influence of calcination temperature and reaction atmosphere on the catalytic properties of Pt-Sn/SAPO-34 for propane dehydrogenation. *Chin. J. Catal.* **31**, 552–556 (2010).
19. Shi, L. *et al.* Al_2O_3 Nanosheets Rich in Pentacoordinate Al^{3+} Ions Stabilize Pt-Sn Clusters for Propane Dehydrogenation. *Angew. Chem. Int. Ed.* **54**, 13994–13998 (2015).
20. Duan, Y., Zhou, Y., Zhang, Y., Sheng, X. & Xue, M. Effect of sodium addition to PtSn/AlSBA-15 on the catalytic properties in propane dehydrogenation. *Catal. Letters* **141**, 120–127 (2011).
21. Zhang, Y., Zhou, Y., Huang, L., Xue, M. & Zhang, S. Sn-modified ZSM-5 As support for platinum catalyst in propane dehydrogenation. *Ind. Eng. Chem. Res.* **50**, 7896–7902 (2011).

22. Zhang, Y. *et al.* Structure and catalytic properties of the Zn-modified ZSM-5 supported platinum catalyst for propane dehydrogenation. *Chem. Eng. J.* **270**, 352–361 (2015).

23. Zhang, Y. *et al.* Comparative study of bimetallic Pt-Sn catalysts supported on different supports for propane dehydrogenation. *J. Mol. Catal. A Chem.* **381**, 138–147 (2014).

24. Fan, X. *et al.* Dehydrogenation of propane over PtSnAl/SBA-15 catalysts: Al addition effect and coke formation analysis. *Catal. Sci. Technol.* **5**, 339–350 (2015).

25. Ren, G. Q. *et al.* Effect of group IB metals on the dehydrogenation of propane to propylene over anti-sintering Pt/$MgAl_2O_4$. *J. Catal.* **366**, 115–126 (2018).

26. Zhu, H. *et al.* Sn surface-enriched Pt-Sn bimetallic nanoparticles as a selective and stable catalyst for propane dehydrogenation. *J. Catal.* **320**, 52–62 (2014).

27. Li, B., Xu, Z., Chu, W., Luo, S. & Jing, F. Ordered mesoporous Sn-SBA-15 as support for Pt catalyst with enhanced performance in propane dehydrogenation. *Chin. J. Catal.* **38**, 726–735 (2017).

28. Shan, Y. L., Wang, T., Sui, Z. J., Zhu, Y. A. & Zhou, X. G. Hierarchical $MgAl_2O_4$ supported Pt-Sn as a highly thermostable catalyst for propane dehydrogenation. *Catal. Commun.* **84**, 85–88 (2016).

29. Rochlitz, L. *et al.* Silica-supported, narrowly distributed, subnanometric Pt–Zn particles from single sites with high propane dehydrogenation performance. *Chem. Sci.* **11**, 1549–1555 (2020).

30. Sun, C. *et al.* A comparative study on different regeneration processes of Pt-Sn/γ-Al_2O_3 catalysts for propane dehydrogenation. *J. Energy Chem.* **27**, 311–318 (2018).

31. Waku, T., Biscardi, J. A. & Iglesia, E. Active, selective, and stable Pt/Na-[Fe]ZSM5 catalyst for the dehydrogenation of light alkanes. *Chem. Commun.* **3**, 1764–1765 (2003).

32. Tripathi, S. N. & Chandrasekharaiah, M. S. Thermodynamic properties of binary alloys of platinum metals II: Ir-Pt system. *J. Less-Common Met.* **91**, 251–260 (1983).

33. Ellner, M. Zusammenhang zwischen strukturellen und thermodynamischen eigenschaften bei phasen der Cu-Familie in den T10-B3-systemen. *J. Less-Common Met.* **60**, 15–39 (1978).

34. Massara, R. Le syst6me binaire Pt-Sn. *J. Alloys Compd.* **215**, 175–179 (1994).

35. Lin, C. F., Mohney, S. E. & Y. A. Chang. Phase equilibria in the Pt-In-P. *J. Appl. Phys.* **74**, 4398–4402 (1993).

36. Ellner, M. Zusammenhang zwischen strukturellen und thermo-dynamischen eigenschaften bei phasen der kupfer-familie in T^{10}-B^4-systemen. *J. Less-Common Met.* **78**, 21–32 (1981).

37. Sattler, J. J. H. B. *et al.* Platinum-promoted Ga/Al_2O_3 as highly active, selective, and stable catalyst for the dehydrogenation of propane. *Angew. Chem. Int. Ed.* **53**, 9251–9256 (2014).

38. Im, J. & Choi, M. Physicochemical Stabilization of Pt against Sintering for a Dehydrogenation Catalyst with High Activity, Selectivity, and Durability. *ACS Catal.* **6**, 2819–2826 (2016).

39. Xu, Y. *et al.* Sintering-Resistant Pt on Ga_2O_3 Rods for Propane Dehydrogenation: The Morphology Matters. *Ind. Eng. Chem. Res.* **57**, 13087–13093 (2018).

40. Gao, H. DFT study of the adsorption properties of single Pt, Pd, Ag, in and Sn on the γ-Al_2O_3 (110) surface. *Chem. Phys. Lett.* **657**, 11–17 (2016).

41. Jablonski, E. L., Castro, A. A., Scelza, O. A. & De Miguel, S. R. Effect of Ga addition to Pt/Al_2O_3 on the activity, selectivity and deactivation in the propane dehydrogenation. *Appl. Catal. A: Gen.* **183**, 189–198 (1999).

42. Siddiqi, G., Sun, P., Galvita, V. & Bell, A. T. Catalyst performance of novel Pt/Mg(Ga)(Al)O catalysts for alkane dehydrogenation. *J. Catal.* **274**, 200–206 (2010).
